# The molecular epidemiology of HIV-1 in the Comunidad Valenciana (Spain): analysis of transmission clusters

**DOI:** 10.1038/s41598-017-10286-1

**Published:** 2017-09-14

**Authors:** Juan Ángel Patiño-Galindo, Manoli Torres-Puente, María Alma Bracho, Ignacio Alastrué, Amparo Juan, David Navarro, María José Galindo, Dolores Ocete, Enrique Ortega, Concepción Gimeno, Josefina Belda, Victoria Domínguez, Rosario Moreno, Fernando González-Candelas

**Affiliations:** 1 Unidad Mixta Infección y Salud Pública FISABIO-CSISP/Universidad de Valencia-I2SysBio, Valencia, 46180 Spain; 2 CIBER of Epidemiology and Public Health, Instituto de Salud Carlos III, Madrid, 28029 Spain; 3 Unidad Prevención del SIDA y otras ITS, Valencia, 46017 Spain; 4 Hospital Clínico Universitario, Valencia, 46010 Spain; 5 Dpto. Microbiología, Universidad de Valencia, 46080 Valencia, Spain; 6 Consorcio Hospital General Universitario, Valencia, 46014 Spain; 7 Unidad Prevención del SIDA y otras ITS, Alicante, 03010 Spain; 8 Hospital General Universitario, Castello, 12004 Spain

## Abstract

HIV infections are still a very serious concern for public heath worldwide. We have applied molecular evolution methods to study the HIV-1 epidemics in the Comunidad Valenciana (CV, Spain) from a public health surveillance perspective. For this, we analysed 1804 HIV-1 sequences comprising protease and reverse transcriptase (PR/RT) coding regions, sampled between 2004 and 2014. These sequences were subtyped and subjected to phylogenetic analyses in order to detect transmission clusters. In addition, univariate and multinomial comparisons were performed to detect epidemiological differences between HIV-1 subtypes, and risk groups. The HIV epidemic in the CV is dominated by subtype B infections among local men who have sex with men (MSM). 270 transmission clusters were identified (>57% of the dataset), 12 of which included ≥10 patients; 11 of subtype B (9 affecting MSMs) and one (n = 21) of CRF14, affecting predominately intravenous drug users (IDUs). Dated phylogenies revealed these large clusters to have originated from the mid-80s to the early 00 s. Subtype B is more likely to form transmission clusters than non-B variants and MSMs to cluster than other risk groups. Multinomial analyses revealed an association between non-B variants, which are not established in the local population yet, and different foreign groups.

## Introduction

Of the four phylogenetic groups which comprise HIV-1 (M, N, O, P), group M is the causal agent of the AIDS pandemic^1,2^. The latest UNAIDS/WHO report^3^ estimated in almost 37 million the number of persons infected with HIV globally, with approximately 2.1 million new infections in 2015.

Although the rate of new HIV diagnosis has stabilized from the early 2000s in the European Union and European Economic Area (EU/EEA), transmissions among men who have sex with men (MSM) have experienced a sustained increase, thus following a different trend to other risk groups^4^. This is evident in Spain: during the late 90 s, most HIV new diagnoses were associated to intravenous drug use (IDU). However, in 2013, 51% of the infections occurred among MSM^5,6^. The increasing incidence among MSM is remarkably high in the age range 20–35 years^6^. HIV infections in Spain also affect the foreign population disproportionally: in 2012, 35% of the new diagnosis corresponded to immigrants or persons of foreign origin^6^.

There exist nine subtypes (denoted as A, B, C, D, F, G, H, J and K) and at least 61 circulating recombinant forms (CRFs) within HIV-1 group M^7^. There are differences among HIV-1 variants in several biological features. For instance, some subtypes and CRFs are associated to a faster progression to AIDS than others^8,9^. Genetic and antigenic differences among HIV-1 subtypes and CRFs are also a challenge for the development of an effective HIV-1 vaccine^10^.

Worldwide, the most prevalent HIV-1 subtype is C, accounting for around 50% of all cases. However, the HIV epidemic in Europe, particularly among MSM, is mainly driven by subtype B^11^, with frequent reported transmission clusters affecting this group^12–20^. However, there is evidence for an increased introduction of non-B subtypes^11^. For instance, in a sample of 206 patients from Spain, Abecasis and colleagues^11^ found that CRF02_AG was the second most prevalent HIV-1 variant after subtype B (prevalence <2%).

The Comunidad Valenciana (CV) is the fourth largest region in Spain (~5 million inhabitants), representing >10% of the total population. Genotypic tests of resistance to antiviral drugs are performed routinely for the design of individualized antiretroviral treatments. Here, we have analysed more than 1,800 HIV-1 *pol* sequences obtained between 2004 and 2014 from different patients in the CV. We have used molecular phylogenetic analyses to complement HIV surveillance tasks of the Public Health Directorate of the CV Regional Government. Specifically, we have used this information to infer the distribution of HIV-1 subtypes, to analyse the introductions (and further local expansion) of this virus in the CV, to identify the emergence of new viral variants in this region, and also to analyse which risk groups are currently more vulnerable to HIV infection. The results obtained from this work may be useful in establishing and reinforcing preventive measures in specific target groups.

## Results

Of the 1804 sequences analysed, 1512 (83.8%) belonged to subtype B. Among the 292 non-B sequences, the most prevalent HIV-1 variant was CRF02_AG (n = 66, overall prevalence = 3.7%), followed by subtypes A1, F1 (both n = 34, 1.9%), CRF14_BG (n = 28, 1.6%) and subtype G (n = 20, 1.1%). Other variants (n = 110) were present with a prevalence lower than 1.0%. Considering those patients for whom epidemiological information was available, 83.04% (930/1120) were male vs 16.96% (190/1120) female; 67.12% (637/949) were native from Spain (determined by place of birth) vs 32.87% (312/949) immigrants or of foreign origin; 66.84% (637/953) were MSM, 21.41% (204/953) heterosexual (HT) and 11.54% (110/953) IDUs. One patient was infected vertically and other one was haemophiliac. The mean age was 34.82 years (range 0 to 76) (Table 1). MSMs were more likely to be Spaniards than non-MSM (Fisher’s Exact test, FET: p-value = 5.5 × 10^−5^, odds-ratio, OR = 1.84 (1.35–2.48).

**Table 1:** Distribution of HIV cases in the dataset (n = 1804) classified by viral subtype, gender, nationality, risk group, age and clustering status.

	**B (n** = **1512)**	**A1 (n** = **34)**	**F1 (n** = **34)**	**G (n** = **20)**	**CRF02_AG (n** = **66)**	**CRF14_BG (n** = **28)**	**Others (n** = 110)*	**Total (n** = **1804)**
**Gender**								
Male	818	10	13	5	24	11	49	930
Female	115	6	6	8	25	6	24	190
UNK	579	18	15	7	17	11	37	684
**Geographical origin**								
Spain	579	6	5	3	11	8	25	637
W. Europe and N. America	27	0	0	0	1	0	1	29
Eastern Europe	17	6	5	0	0	5	6	39
Africa and M. East	13	3	3	10	26	0	12	67
Latin America	141	1	3	0	7	3	19	174
Others	1	0	0	0	0	0	2	3
UNK	734	18	18	7	21	12	45	855
**Risk group**								
HT	124	8	6	9	29	2	26	204
MSM	586	3	6	0	11	1	30	637
IDU	85	3	0	2	3	12	5	110
Other	2	0	0	0	0	0	0	2
UNK	715	20	22	9	23	13	49	851
**Clustering**								
No cluster	622	22	24	13	32	5	49	767
Small cluster (2–3)	371	12	5	7	25	2	39	461
Medium cluster (4–9)	240	0	5	0	9	0	22	276
Large cluster (> = >=10)	279	0	0	0	0	21	0	300
**Age: mean (min**–**max)**	35.25 (14–76)	32.8 (19–56)	33.5 (18–62)	28.9 (21–41)	30.6 (19–49)	38.11 (22–65)	33.2 (0–62)	34.82 (0–76)

*The “Others” subset includes 10 subtype C, 1 subtype D, 13 CRF19_cpx, 12 CRF12_BF, 9 CRF06_cpx, 7 CRF47_BF and 58 other (mostly unassigned) recombinant sequences.

**Figure 1:**
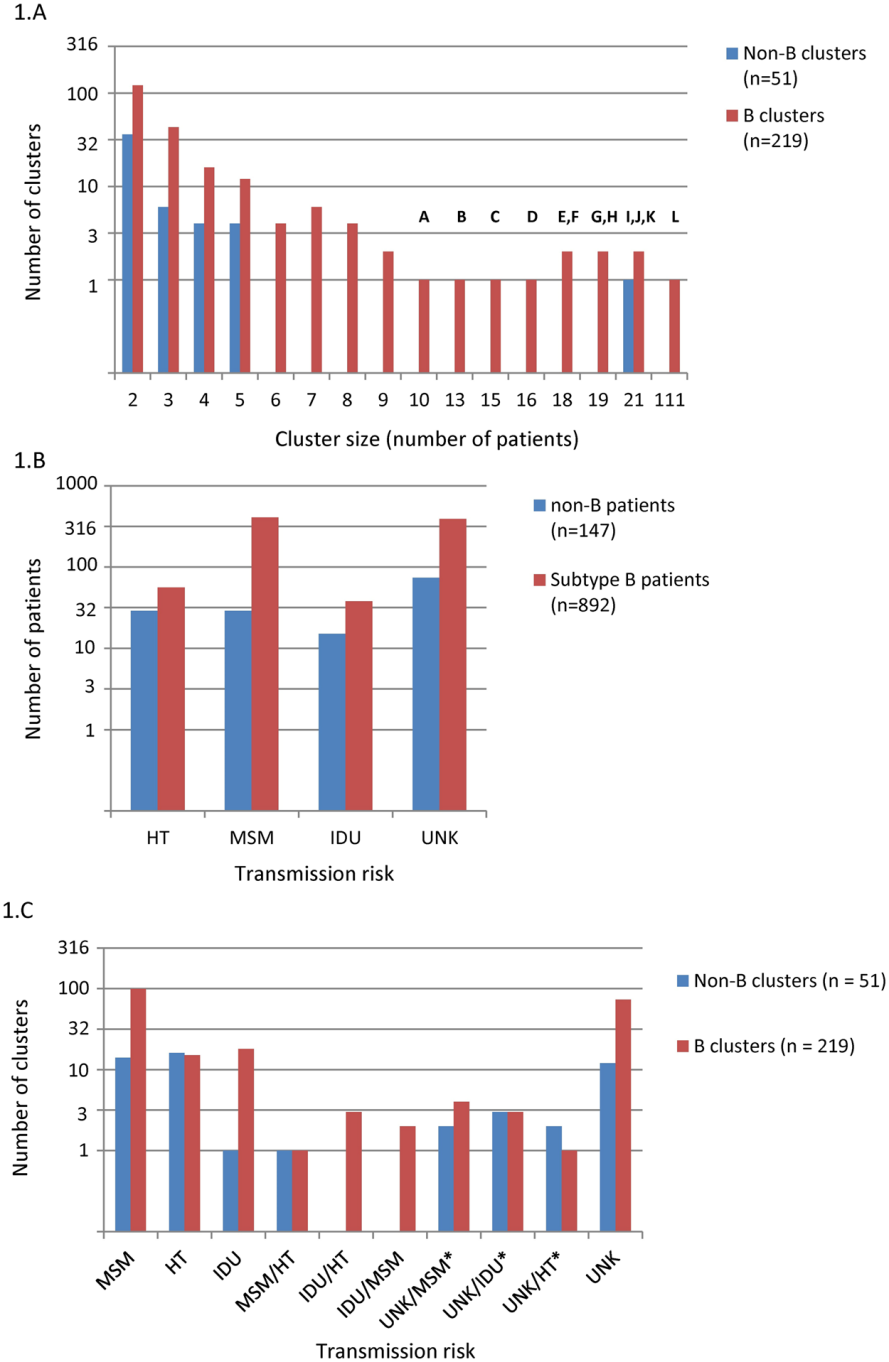
(**A**) Distribution of sizes (log10 scale) of the 270 transmission clusters found in the CV (2004–2014) through phylogenetic analysis. Block letters indicate the 12 clusters that were analysed with BEAST. (**B**) Total number of patients for each risk group included in transmission clusters (n = 1039). (**C**) Number of transmission clusters depending on the risk group in which they were classified. *>1/4 patients shared a known risk group, but they were not enough to classify the cluster.

Phylogenetic analyses revealed the existence of 270 transmission clusters, with sizes ranging from 2 to 111 patients (Fig. 1A). In total, 1039 patients from the dataset were included in a transmission cluster (57.5%), with 302 patients (16.7%) included in large clusters of 10 or more patients (Table 1; Fig. 1A). Among the 892 patients clustering in transmission groups of subtype B, 407 (45.6%) were MSM, 56 (6.3%) HTs, 38 (4.2%) IDUs and 391 (43.8%) of unknown transmission route. On the other hand, of the 147 patients clustering in non-B clusters 29 (19.7%) were HTs, 29 (19.7%) MSM, 15 (10.2%) IDUs and (50.3%) 74 of unknown transmission route (Fig. 1B). Among the transmission clusters that could be classified by the most frequent risk of transmission (≥50%) for the CV patients included in them, the most prevalent transmission clusters of subtype B were those classified as MSM (n = 99/219, 45.2%), followed by IDUs (n = 18/219, 8.2%) and HTs (n = 15/219, 6.8%). However, for non-B transmission clusters, ﻿thos﻿e of HTs were the most frequent ones (n = 16/51, 31.4%), followed by MSM (n = 14/51, 27.5%) and IDUs (n = 1/51, 2.0%). 87 of the 219 (39.7%) subtype B clusters and 20 of 51 (39.2%) non-B clusters could not be classified based on a majority risk of transmission (Fig. 1C). Subtype B sequences were more likely to be part of a transmission cluster than non-B sequences (FET: p-value = 0.007, OR = 1.42, 95% CI = 1.09–1.84). Also, MSM were more likely to be part of a transmission cluster than other risk groups (FET: p-value = 1.68 × 10^−4^, OR = 1.56, 95% CI = 1.23–1.99).

**Figure 2:**
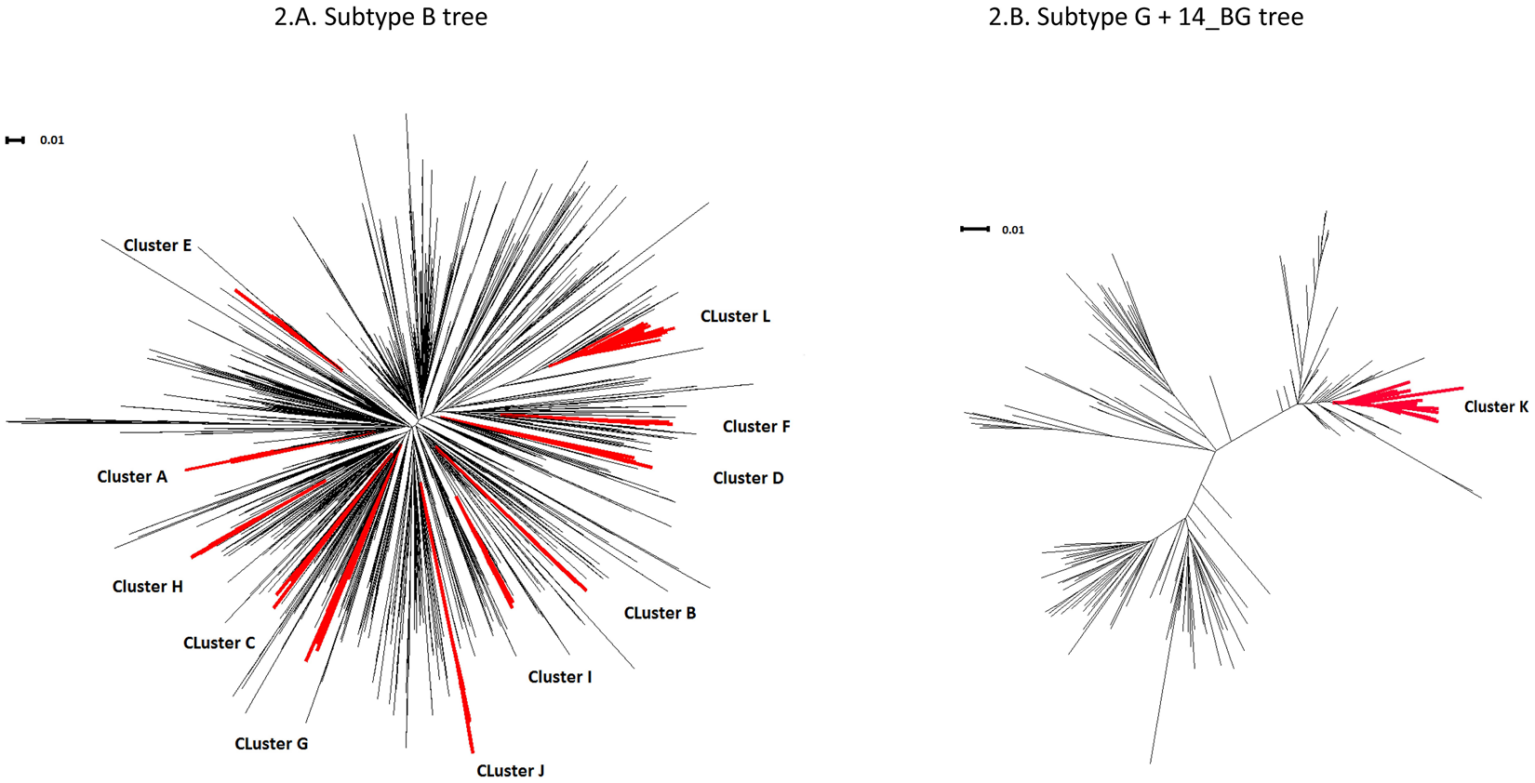
Maximum likelihood trees with the 12 largest transmission clusters highlighted in red. (**A**) Subtype B tree (clusters A-J, L). (**B**) Subtype G and CRF14_BG tree (cluster K).

**Figure 3:**
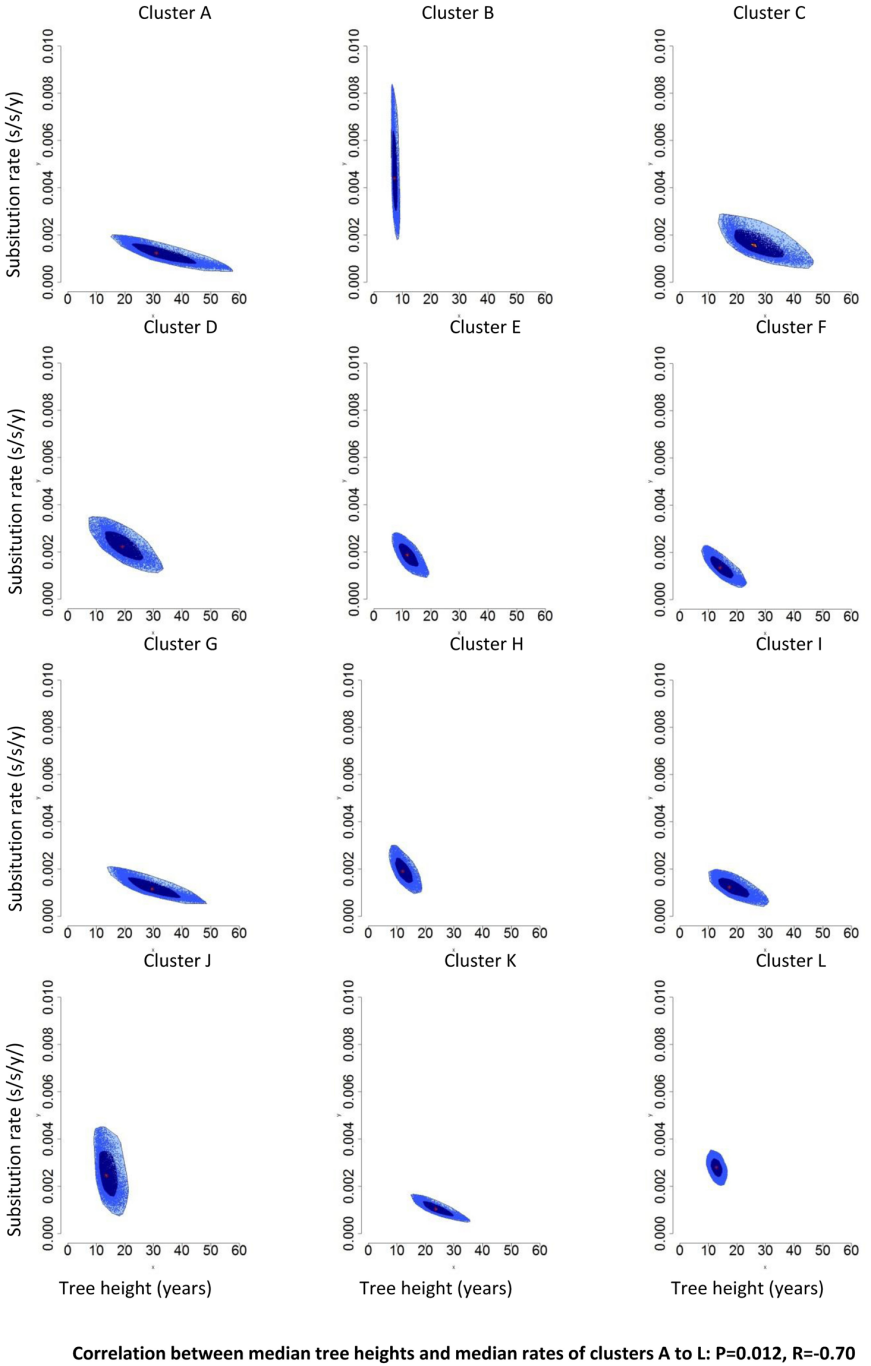
Bagplots representing tree height and evolutionary rate estimates, obtained from the posterior distribution of the 12 largest transmission clusters detected (**A** to **L**).

Twelve large transmission clusters, which included at least 10 patients from the CV (Fig. 2), were detected: 11 were from subtype B and included a total of 281 patients (Fig. 2A). One cluster of 21 patients corresponded to CRF14_BG (Cluster K, Fig. 2B). Patients infected with subtype B were more likely to form large transmission clusters than those infected with non-B variants (FET: p-value = 3.4 × 10^−7^, OR = 2.94, 95% CI = 1.84–4.92). Nine of the large clusters were classified as MSM; the other three included mostly IDUs, although in a proportion lower than 50% (Table 2). Dated phylogenies of these clusters (Supplementary Figs 1 to 12) revealed that they originated between 1984 (cluster G, unclassified transmission route) and 2005 (cluster B, MSM). Only transmission clusters that included MSM as the main transmission risk were originated since 2000 (Table 2; Supplementary Figs 1 to 12). Bagplots (bivariate representations of boxplots^21^) representing tree height (the time elapsed between tMRCA and the sampling date of the last sequence) and evolutionary rate estimates from the posterior distribution of each large cluster are shown in Fig. 3. A significant, negative correlation between median tree height and substitution rate estimates of these large clusters was obtained (R = −0.70, p-value = 0.013).

**Table 2:** Size (n, number of patients), risk group, range of sampling dates and root-to-tip vs sampling date correlation coefficient for each transmission cluster (R), and estimates of their tMRCAs (median) and substitution rates as obtained with BEAST under the best-fitting demographic model (EXPO: exponential growth; BSP: Bayesian Skyline Plot; LOG: logarithmic growth).

Transmission cluster	n	risk group	range #	R	Model	tMRCA (95% HPD)#	Substitution rate (95% HPD)*
A	10	MSM	2004–2011	(−0.08)	EXPO	1984.7 (1971.2–1994.5)	0.0014 (0.0009–0.0021)
B	13	MSM	2007–2013	0.61	BSP	2005.9 (2003.8–2006.9)	0.0043 (0.0016–0.0087)
C	15	UNK/IDU	2004–2014	(−0.18)	BSP	1988.0 (1969.0–1999.2)	0.0015 (0.0007–0.0025)
D	16	MSM	2011- 2013	(−0.05)	LOG	1994.9 (1982.4–2006.3)	0.0022 (0.0012–0.0034)
E	18	UNK/MSM	2010–2014	0.25	EXPO	2002.2 (1995.9–2006.8)	0.0018 (0.0011–0.0027)
F	18	MSM	2008–2013	0.40	LOG	1999.3 (1989.6–2005.0)	0.0013 (0.0005–0.0023)
G	19	UNK	2004–2013	(−0.05)	EXPO	1984.3 (1966.9–1997.0)	0.0011 (0.0006–0.0019)
H	19	MSM	2008–2014	0.58	BSP	2002.1 (1995.9–2006.2)	0.0019 (0.0010. 0.0029)
I	21	MSM	2004–2012	0.58	LOG	1994.6 (1982.2–2001.3)	0.0012 (0.0005–0.0020)
J	21	MSM	2004–2013	0.76	LOG	1999.5 (1993.2–2003.4)	0.0023 (0.0010–0.0045)
K	21	UNK/IDU	2004–2014	0.62	BSP	1990.6 (1979.6–1998.1)	0.0010 (0.0005–0.0016)
L	111	MSM	2006–2014	0.61	EXPO	2001.8 (1998.6–2004.8)	0.0028 (0.0022–0.0035)

^#^Time measured in years. *Substitutions per site and year.

Despite most patients infected with subtype B were Spanish natives (579 Spaniards vs 199 foreigners), a majority of those infected with non-B variants were foreigners (58 vs 113; FET: p-value < 2.2 × 10^−16^, OR = 5.66, 95% CI = 3.91–8.23). This difference was also observed when considering only patients belonging to a transmission cluster (subtype B: 378 vs 114; non-B: 31 vs 47; FET: p-value = 1.8 × 10^−10^; OR = 5.01, 95% CI = 2.96–8.57) (Supplementary Table S1).

A total of 88 subtype B, and 9 non-B transmission clusters did not include patients of foreign origin. These subtype B clusters included 178 Spanish patients and 122 of unknown origin, and the non-B clusters included 13 Spanish patients and 14 of unknown origin. A total of 49 subtype B, and 7 non-B transmission clusters were formed by both Spanish and non-Spanish patients. Altogether, these mixed subtype B clusters included 200 Spanish, 87 non-Spanish (mostly from Latin America) and 113 of unknown origin, and the mixed non-B clusters included 18 Spanish, 10 non-Spanish (mostly from Latin America or Eastern Europe) and 13 of unknown origin. All large transmission clusters but one (cluster G, for which there was not information available in 17 of its 19 patients) presented this characteristic. Finally, a total of 16 subtype B and 25 non-B transmission clusters included only patients of foreign origin. These subtype B clusters included 27 foreign patients (mostly from Latin America) and 16 of unknown origin, and the non-B clusters included 37 foreign patients (mostly from Africa/Middle East and Latin America) and 20 of unknown origin (Supplementary Table S1).

In addition to the patients’ origin, other variables considered for the multinomial analysis presented differences between subtypes. So, subtype B sequences were disproportionally more present in males compared to females (FET: p-value < 2.2 × 10^−16^, OR = 4.75, 95% CI = 3.29–6.86), in MSM with respect to HTs and IDUs (p-value < 2.2 × 10^−16^, OR = 7.4, 95% CI = 4.87–11.31 and p-value = 2 × 10^−5^, OR = 3.37, 95% CI = 1.90–5.89, respectively), and in IDUs compared to HTs (p-value = 0.0038, OR = 2.19, 95% CI = 1.26–3.88). There were also differences between subtypes with respect to age distribution (Kruskal-Wallis test: P = 0.0013, Chi^2^ = 21.8, df = 6), with CRF02_AG patients being significantly younger than B patients (Games-Howell post-hoc test result for this comparison: p = 0.001, t = 4.37, df = 58). Given that no significant differences were found between subtypes in the distribution of collection dates (P > 0.70), this variable was excluded from the multinomial analysis.

Multinomial analyses were performed using a subset of 906 patients for whom there was information for all the variables considered: sex, risk group, country of origin, clustering status and age. We considered as baseline the most frequent category for each variable (subtype B, Spaniard, male, MSM, age between 21 and 29 years and not clustering), and a significant model was obtained (McFadden R^2^ = 0.32, Chi^2^ = 421.34, p-value < 2.2 × 10^−16)^). We checked that this subset was representative of the global set (n = 1804) by means of FETs. The p-values obtained for all the analysed variants were >0.05, including the distribution of patients from the different cluster-size categories (no cluster, small cluster, medium cluster and large cluster) that would result if only sequences included in the multinomial analysis had been taken into account for cluster detection, (FET: P > 0.10; Supplementary Table S2). This dataset was also genetically representative of the whole dataset (Supplementary Fig. S13). The multivariate model shows that the chances of being infected with all non-B groups increased in foreign patients, coming from Eastern Europe (A1, F1, 14_BG and the pooled, rare, variants), Africa and the Middle East (F1, G, 02_AG and the rare variants) and Latin America (rare variants), with all p-values < 0.01, all ORs >3.0. The likelihood of being infected with CRF14_BG also increased in patients older than 50 years (p-value = 0.029, OR = 36.2, 95% CI = 1.45–904.0), in IDUs (p-value = 9.44 × 10^−5^, OR = 257, 95% CI = 15.8–4160) and in those patients forming large transmission clusters (p-value = 1.6 × 10^−4^, OR = 36.9, 95% CI = 5.67–240) (Table 3).

**Table 3:** Results of the multinomial analysis (only significant associations between each HIV variant and the categories compared at each variable are shown).

Coefficients								
	Estimate	Std error	t-value	Pr(>|t|)		OR	2.50%	97.50%
14_BG:Age(>50)	3.59	1.64	2.188	0.028696	*	36.2	1.45	904
14_BG:RISK(HT)	2.39	1.43	1.674	0.094265	$	11.0	0.664	181
02_AG:RISK(HT)	1.07	0.579	1.850	0.064773	$	2.91	0.937	9.07
14_BG:RISK(UDI)	5.55	1.42	3.905	9.44E-05	***	257.0	15.8	4160
14_BG:Eastern Europe	3.29	1.09	3.023	0.002502	**	26.9	3.18	227
F:Eastern Europe	3.51	1.08	3.250	0.001155	**	33.5	4.03	279
OTHERS: Eastern Europe	2.01	0.583	3.448	0.000566	***	7.47	2.38	23.5
A1:Eastern Europe	2.78	0.795	3.493	0.000478	***	16.1	3.38	76.3
F1:Africa and Middle East	3.50	1.05	3.353	0.000799	***	33.3	4.29	258
G:Africa and Middle East	3.78	1.17	3.236	0.001218	**	43.7	4.43	431
OTHERS: Africa and Middle East	2.77	0.554	5.009	5.48E-07	***	16.0	5.41	47.4
A1:Africa and Middle East	1.92	0.993	1.932	0.053351	$	6.81	0.973	47.7
02_AG:Africa and Middle East	3.76	0.580	6.478	9.28E-11	***	42.9	13.8	134
14_BG:Latin America	2.71	1.38	1.955	0.050547	$	15.0	0.994	225
OTHERS:Latin America	1.19	0.352	3.391	0.000696	**	3.30	1.66	6.58
14_BG:ClusterLARGE	3.61	0.955	3.776	0.000159	***	36.9	5.67	240

***P < 0.001; **0001 < P < 0.01; *0.01 < P < 0.05; $0.05 < P < 0.

**Figure 4:**
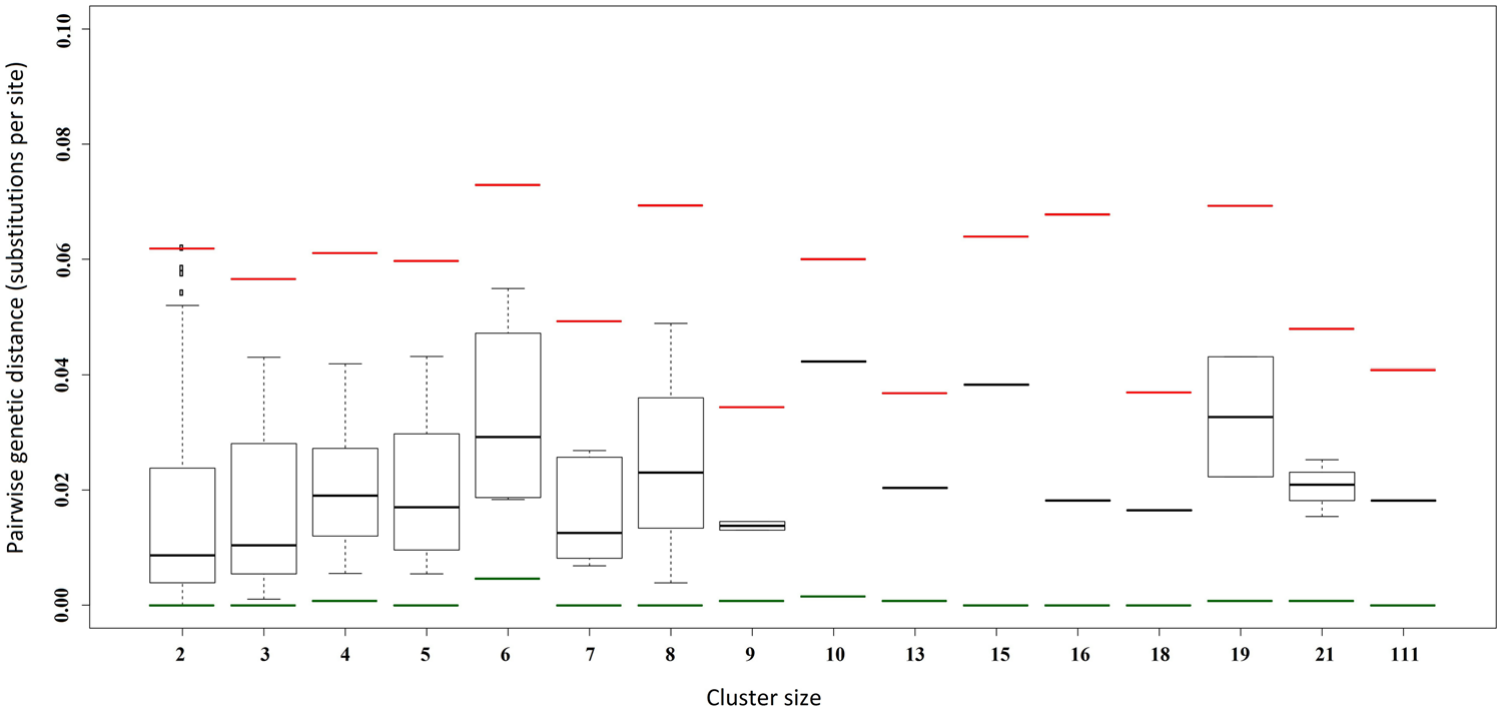
Boxplots representing mean pairwise genetic distances (s/s) between individuals within transmission clusters of different sizes, as estimated with the TN93 + GAMMA (4 CAT) model. Green and red bars represent the minimum and maximum pairwise distances, respectively, between a pair of individuals from a given cluster size.

Pairwise genetic distances (GD) between individuals from a given transmission cluster ranged between 0.00 substitutions/site (s/s), in clusters of sizes of 2, 3, 5, 7, 8, 15, 16, 18 and 111 individuals, and 0.073 s/s, in a cluster of 6 individuals. For most pairwise comparisons, GD was lower than 0.045 s/s, regardless the size of the transmission cluster (Fig. 4; Supplementary Table S3).

## Discussion

We have studied the HIV epidemic in the Comunidad Valenciana by analysing, with molecular and evolutionary tools, 1804 sequences obtained between 2004 and 2014 from different patients. Our results indicate that the HIV epidemic in the CV is dominated by HIV-1 subtype B infections among local MSM. However, non-B infections represented an important number of cases, with a prevalence higher than 15%, being CRF02_AG the most prevalent non-B variant (prevalence = 3.7%), in agreement with previous estimates for Spain^11,22^.

Overall, the detection of transmission clusters demonstrates the importance of the domestic spread of HIV-1 in the CV. Most patients from the whole dataset (>57%) were included in local transmission clusters, of sizes ranging from 2 to 111 individuals. Local transmission was especially important among MSM, who were more likely to be included in a transmission cluster, as well as for Spanish natives, than other risk groups. Considering the 12 largest clusters (size ≥10 patients), this transmission risk was the most frequent in 9 of them. Excluding transmission cluster A (MSM, tMRCA = 1984.7), MSM clusters were of more recent origin as estimated using Bayesian coalescent analyses, especially clusters B (n = 13), E (n = 18), H (n = 19) and L (n = 111), which originated after year 2000. Previous analyses in the Spanish regions of Madrid^23^ and the Basque Country^24^ detected lower proportions of clustering patients (18 and 27% of their analysed sequences, respectively). These results suggest that the importance of local transmissions may not be the same in different Spanish regions. In addition, they also found evidences for an increased vulnerability to HIV of the Spanish MSM community in recent years. A more detailed analysis of cluster L is provided elsewhere^25^.

Immigrants were disproportionally represented in the dataset (almost 1/3 of the patients were of non-Spanish origin), reflecting their higher vulnerability to HIV infection. Multinomial analyses evidenced the significant association between all non-B groups analysed and different foreign populations: Eastern Europe (subtypes A1, F1, CRf14_BG and the rare variants), Africa and the Middle East (subtypes F1, G, CRF02_AG and rare variants) and Latin America (rare variants). These associations were in agreement with the geographical distributions of these variants^26–28^. These results, along with the fact that most non-B patients, either clustering or not, were of non-Spanish origin (CRF14_BG was the only exception) suggest that along the analysed time-span non-B HIV variants were not well established among Spanish locals in this region. This is exemplified by the fact that almost half of the non-B transmission clusters did not include any Spanish patient. Furthermore, non-B patients clustered in a significantly lower proportion than patients infected with subtype B, especially when considering clusters with at least 10 patients, thus displaying significantly lower local transmission efficiency. Previous molecular epidemiology studies of HIV-1 in Western Europe also reported non-B variants to be associated with the migrant population^11^, mainly affecting HTs. In the Spanish region of Madrid, where non-B variants have been found with a prevalence of up to 37%^23^, more than 70% of non-B sequences were found to belong to non-Spanish patients^29^. Other works in different Western European countries have reported differences in the prevalence of non-B variants in the migrant population. Similarly to our results, in countries such as Switzerland (estimated prevalence of non-B HIV sequences = 21%^30^) or the Belgian-Luxemburg population (prevalence >45%^11,31^) nearly 70% of non-B variants have been associated to patients of migrant origin^30,31^. These results contrast with those from Portugal, the Western European country where subtype B displays the lowest prevalence^11^. An study in the Portuguese region of Minho (close to the border with Spain) found non-B variants to have a prevalence of 73%, and only 10% of these sequences belonged to non-Portuguese patients^32^, thus evidencing that non-B infections are already established among locals.

Although 11 of the 12 largest transmission clusters were of subtype B, one of the largest clusters found (n = 21, median tMRCA = 1990.6) corresponded to the CRF14_BG, and included a high number of IDUs from Spain. CRF14 has been associated to a predominance of CXCR4 tropism, which usually leads to faster AIDS onset^33,34^. Previous phylogeographic analyses have suggested that CRF14_BG originated in the Iberian Peninsula^33^, and its prevalence is increasing in some Eastern European countries, such as Romania, boosted by migration between these countries and Spain^35^. Similarly to Romania, this CRF has been found to be common among IDUs in Greece, where a large transmission network of CRF14_BG of recent origin (tMRCA = 2009) has been reported to affect more than 70 people, and which origin has been linked to strains from South-western Europe^36^.

We also detected two other transmission clusters of smaller size (n = 5 and n = 4) affecting Spanish-native MSMs of another highly pathogenic variant (CRF19_cpx), which we previously reported as the first evidence of expansion of this variant outside Cuba^37^. Despite the prevalence of these CRFs remaining low in the CV, the effective expansion of these highly pathogenic HIV variants, evidenced by the detection of transmission clusters, is of especial interest because it may hamper the control of the local HIV epidemic, especially among vulnerable populations such as IDUs or MSMs.

The significant association between non-B variants and immigrants reported in this work and in previous molecular epidemiology analyses of HIV-1 in Madrid, Spain^23,29^, as well as the aforementioned examples of emergence of CRFs associated with migratory waves, suggest that high migration and tourism rates in some Spanish regions may contribute to the high genetic diversity of their HIV-1 epidemics. Thus, although our results suggest that non-B HIV groups are not well established in the local population, with only a few of them including patients of both Spanish and non-Spanish origin, this situation could change soon without intensified HIV prevention campaigns focused on vulnerable groups, including migrants, IDUs and MSM.

Although the large number of sequences for which no epidemiological information was available could be a potential limitation of our study, the results reported are representative of the sampled population and represent one of the most comprehensive analysis of the HIV pandemics in Spain^22,23,29,38^.

We have found a significant, negative correlation between tree height and evolutionary rate estimates obtained when comparing the 12 largest clusters. Several publications have addressed this time-dependency on the evolutionary rate (TDRP), that is, virus lineages estimated to have a recent origin yield higher estimated evolutionary rates than older viruses^39–41^. In our study the most plausible explanation for this phenomenon is the overestimation of evolutionary rates in the most recent clusters, caused by the presence of deleterious mutations over which purifying selection has not had time to act. This phenomenon might be potentiated by the bottlenecks that occur at viral transmission, which might originate a transient accumulation of deleterious mutations. Consequently, these results suggest that TDRP is an important factor to consider in molecular epidemiology, even when datasets are obtained from the same population and at short timescales (in this case 10 years, from 2004 to 2014).

It is also important to mention that many works on the molecular epidemiology of HIV-1 remove resistance-associated positions from the analysis, in order to prevent spurious clustering by convergent evolution. In our analyses, we did not remove those positions because their impact on the detection of transmission clusters has been demonstrated to be irrelevant^42^. Furthermore, a great majority of our sequences were known to derive from treatment-naïve patients (only 66 patients were known to have been treated).

Additionally, although the identification or detection of transmission clusters is commonly based only on phylogenetic cluster support, it is also often based on criteria that combine phylogenetic support, usually high bootstrap values, with genetic distance (GD) thresholds^43^. In this work, we did not consider any GD threshold value as criterion to define transmission clusters. This is because the rationale for genetic distance cut-offs is rarely provided, with very different thresholds being reported in the literature^44^, and larger GD values are expected for older transmission events. In this way, choosing distance-based thresholds can lead to underestimating transmission clusters, especially those that span long time periods^43^, as it is the case of our dataset. Nevertheless, a great majority of GDs estimated from the pairwise comparisons performed here were lower than 0.045 s/s. Thus, almost all the transmission clusters reported here would be characterized by both high phylogenetic support and low GD.

In conclusion, our results provide evidence that the HIV-1 epidemic in the CV is dominated by subtype B, especially among local MSMs. Although there was an important number of non-B cases, they occurred mostly among immigrants. This suggests that non-B infections are not well established in the local population. However, the detection of transmission clusters of non-B variants associated to a higher pathogenicity and affecting Spanish patients, urges to increase efforts on HIV testing and prevention campaigns to prevent their further expansion.

## Materials and Methods

### Dataset

A total of 1804 PR/RT sequences were obtained from newly HIV diagnosed people at six different hospitals and two HIV counselling and testing centres (CIPS) from the three provinces in the CV between 2004 and 2014. The sequences comprised the complete PR and the first 1005 nucleotides (335 amino acids) of the RT (1302 nt in total), and were obtained and subtyped as detailed in Patiño-Galindo *et al*.^25^.

### Detection of local transmission clusters

Detection of transmission clusters, was performed following the same two-step procedure detailed in Patiño-Galindo *et al*.^25^. Briefly, in the first step, we obtained independent phylogenetic trees for each HIV-1 subtype and CRF detected, which included reference sequences retrieved from the Los Alamos HIV database spanning the analysed PR/RT region (n = 1787 subtype B, 2097 A1, 720 F1, 854 G + CRF14_BG and 2157 CRF02_AG, as well as 5720 sequences from all the HIV-1 subtypes/CRFs that were rare in the CV dataset). The initial trees were reconstructed with FastTree 2.1 in order to detect potential transmission clusters, defined as clades with SH-like support ≥0.70, and containing ≥90% sequences from the CV dataset^17^.

In the second step, these potential clusters were confirmed with ML phylogenies obtained with PhyML 3.0^45^ in which the following additional reference sequences were included: (i) the 10 sequences with the highest similarity to each CV sequence from the potential clusters, as retrieved with a BLASTN search at the NCBI server (https://blast.ncbi.nlm.nih.gov/Blast.cgi); (ii) 854 subtype B, 77 A1, 74 F1, 173 G + CRF14_BG, 101 CRF02_AG as well as 482 reference sequences from the other HIV-1 subtypes/CRFs. Only those potential clusters which contained more than 90% of sequences from the CV and grouped in the ML tree with aLRT support ≥0.98 were considered as confirmed. Clusters were then classified depending on the major risk of transmission (≥50%) for the corresponding patients from the CV.

### Dated phylogenies

Dated phylogenies of large transmission clusters (those containing at least 10 patients from the CV^46^) were obtained with BEAST v1.8.1^47^, similarly as in Patiño-Galindo *et al*.^25^. In those clusters with low root-to-tip divergence vs sampling date correlation (R < 0.4), a log-normal prior (median = 0.0025 per site and year, s/s/y, 95% HPD upper limit = 0.0035 s/s/y)^17^ was placed on the ucld.mean parameter.

### Statistical analyses

A multinomial logistic regression analysis was performed in order to identify the main predictors of HIV-1 subtype/CRF group distribution, considering relevant epidemiologic variables: country of origin, sex, risk group, collection date, age and clustering status. Due to the lack of epidemiological data for many sequences from the dataset, only 906 of the 1804 sequences were included in this analysis. Seven groups of different HIV-1 subtypes and CRFs were used: A1 (n = 14), B (n = 752), F1 (n = 13), G (n = 11), 02_AG (n = 42) and 14_BG (n = 15). All HIV-1 variants with fewer than 10 patients sampled were pooled as “Other variants” (n = 59). Prior to the multinomial analysis, univariate analyses (Fisher’s Exact Tests) were performed for the aforementioned variables, in order to exclude from the multinomial analysis those with non-significant p-values. Only the variable “collection date” (p-value > 0.70) was excluded. In the multinomial analysis, the most representative category of each variable was used as “baseline category” (Subtype B, Spaniard, male, MSM, age between 21 and 29 years and not clustering; Supplementary Table S4). All the statistical analyses were performed using R^48^. The mlogit R package^49^ was used for the multinomial analysis.

### Estimation of pairwise genetic distances

Pairwise genetic distances (GD) between individuals within transmission clusters were estimated, as substitutions per site (s/s), with the TN93 + Γ(4CAT) model, using Perl and R scripts (available upon request) dependent on the ape R package^50^.

### Sequence information

GenBank accession numbers for the CV sequences used in this study are HF567872-HF567912 and MF403205–MF404967.

## Electronic supplementary material

Supplementary Material
