# Supplementary material for: The molecular epidemiology of HIV-1 in the Comunidad Valenciana (Spain): analysis of transmission clusters
Source: Sci Rep. 2017 Sep 14;7:11584. doi: 10.1038/s41598-017-10286-1 (PMC5599654; doi:10.1038/s41598-017-10286-1)
Supplement: Supplementary file 1 — Supplementary Material [file 41598_2017_10286_MOESM1_ESM.pdf]

Supplementary Material for the Manuscript

The molecular epidemiology of HIV-1 in the Comunidad Valenciana (Spain): analysis of transmission clusters

Juan Ángel Patiño-Galindo [a,b], Manoli Torres-Puente [a], María Alma Bracho [a,b], Ignacio Alastrué [c], Amparo Juan [c], David Navarro [d,e], María José Galindo [d], Dolores Ocete [f], Enrique Ortega [f], Concepción Gimeno [e,f], Josefina Belda [g], Victoria Domínguez [h], Rosario Moreno [h], Fernando González-Candelas [a,b*]

a Unidad Mixta Infección y Salud Pública FISABIO-CSISP/Universidad de Valencia-I2SysBio, Valencia, 46180, Spain

b CIBER of Epidemiology and Public Health, Instituto de Salud Carlos III, Madrid, 28029, Spain

c Unidad Prevención del SIDA y otras ITS, Valencia, 46017, Spain

d Hospital Clínico Universitario, Valencia, 46010, Spain

e Dpto. Microbiología, Universidad de Valencia, 46080, Valencia, Spain

f Consorcio Hospital General Universitario, Valencia, 46014, Spain

g Unidad Prevención del SIDA y otras ITS, Alicante, 03010, Spain

h Hospital General Universitario, Castelló, 12004, Spain

CONTENTS

Supplementary Figures S1-S12. Dated phylogenetic trees of the 12 largest transmission clusters (A to L) analyzed with BEAST. Branch lengths represent years. Nodes with Posterior Probabilities ≥ 0.90 are represented with black dots.

Supplementary Figure S13. Phylogenetic tree obtained with FastTree, including the 1804 CV sequences, and 133 subtype/CRF references from LANL. CV sequences that were included in the multinomial analysis are colored in red.

Supplementary Table S1. Geographical origin of the patients in transmission clusters. Number of clusters, and number of patients (Spanish, non-Spanish, unknown origin), regarding the total number of B and non-B transmission clusters, the 12 large transmission clusters, and the other transmission clusters that include i) both Spanish and non-Spanish patients (mixed clusters, ii) only Spanish patients, iii) only non-Spanish patients, iv) only patients of unknown origin. Non-B mixed clusters are described individually.

Supplementary Table S2. Cluster size distribution, if only patients included in the multinomial analysis were considered.

Supplementary Table S3. Summary of nucleotide divergence estimates among sequences within each cluster. Average, minimum and maximum values of Tamura-Nei estimates of nucleotide divergence are reported for each cluster.

Supplementary Table S4. Distribution of HIV cases in the dataset subjected to multinomial analysis (n=906), regarding the variables subtype, gender, age, geographical origin, risk group and clustering status.

Supplementary Figure S.1 Cluster A

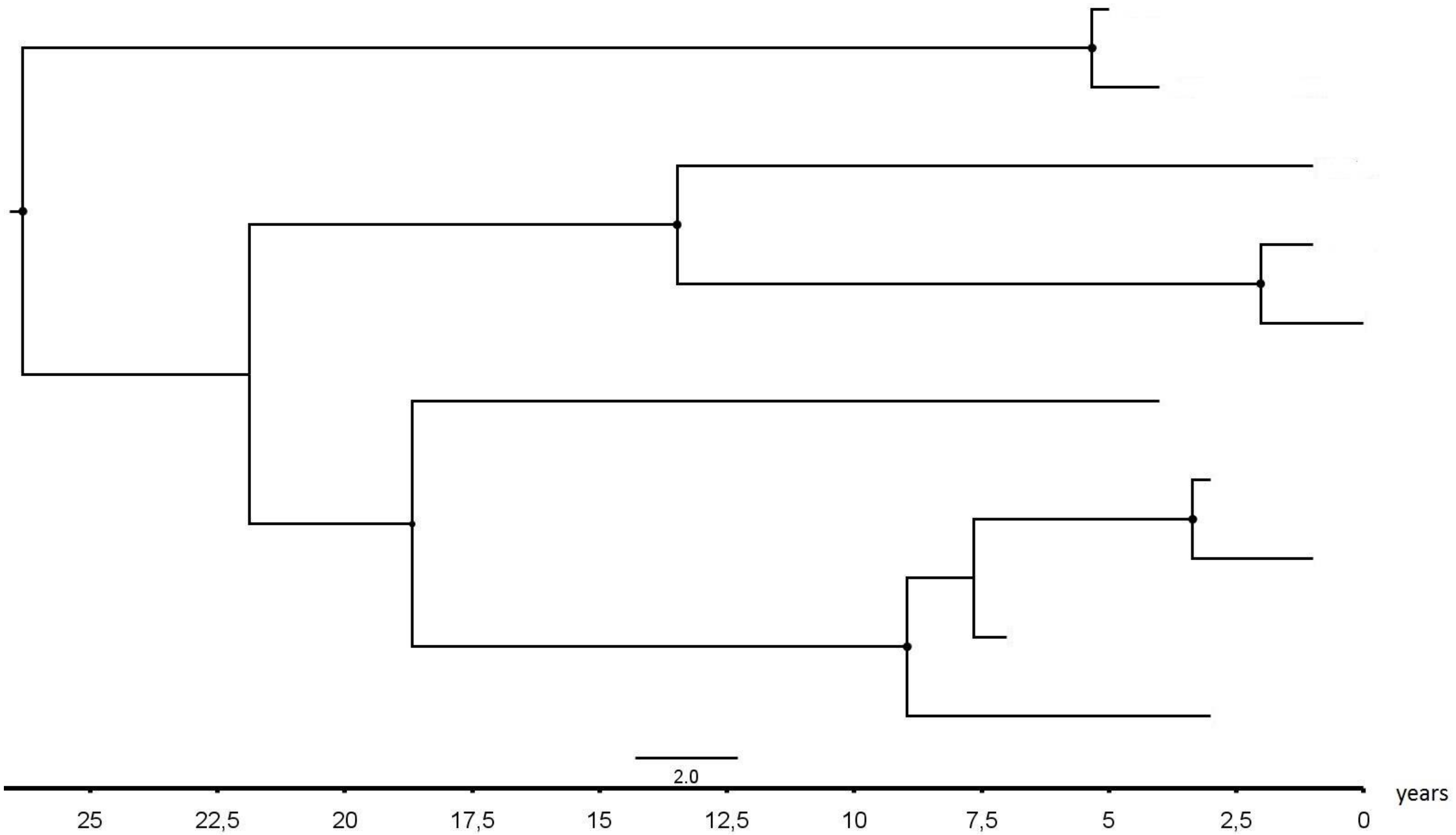

Supplementary Figure S.2 Cluster B

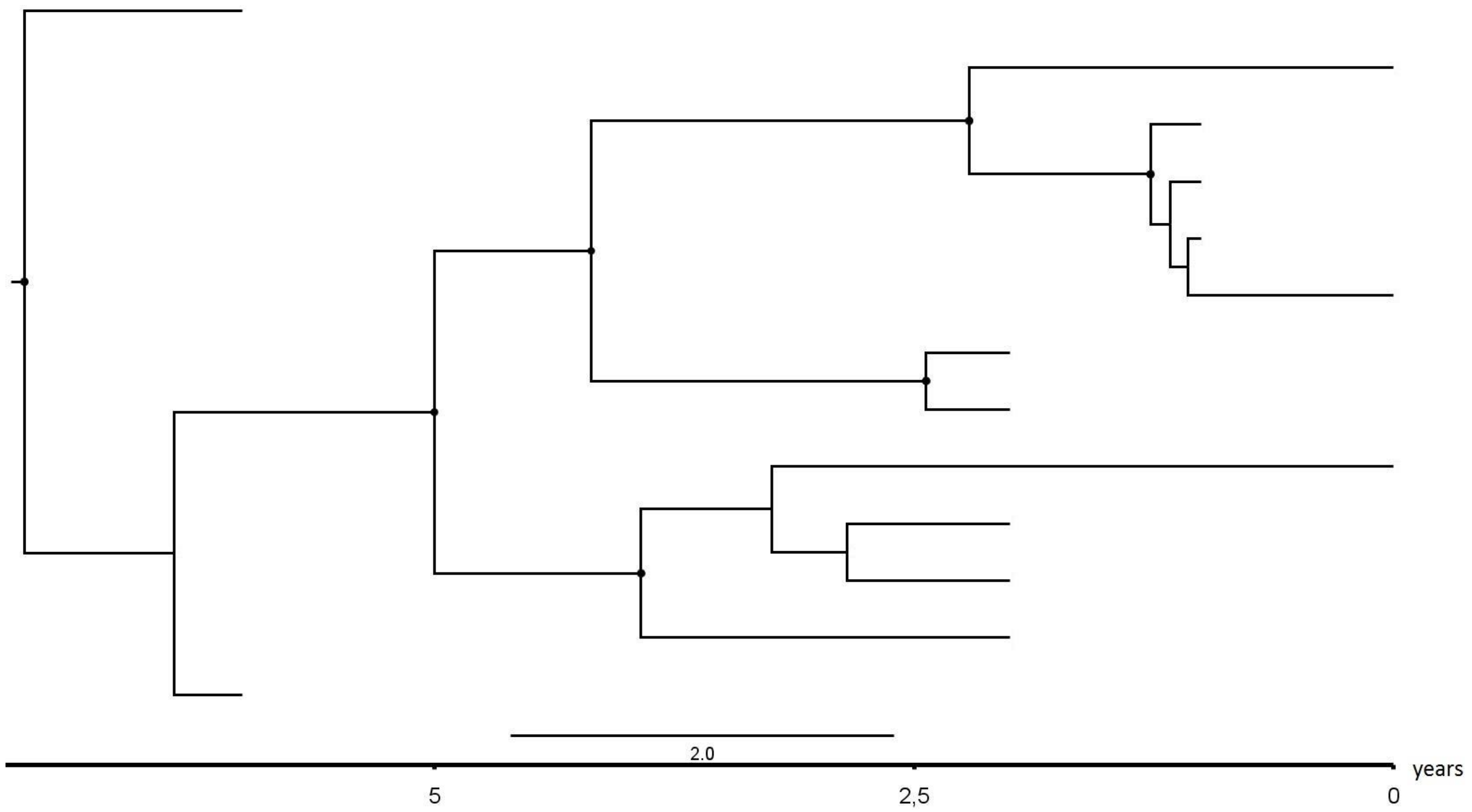

Supplementary Figure S.3 Cluster C

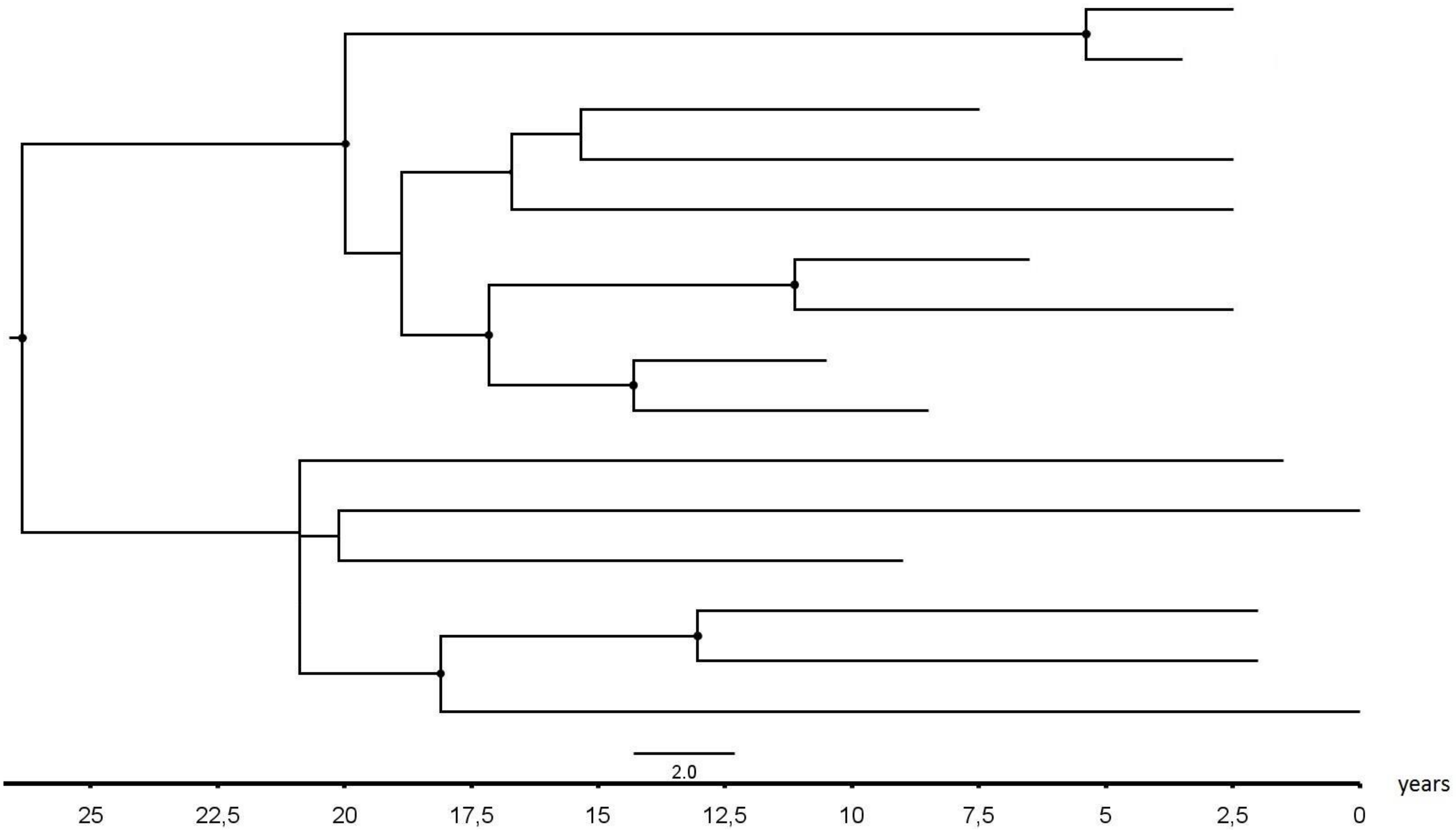

Supplementary Figure S.4 Cluster D

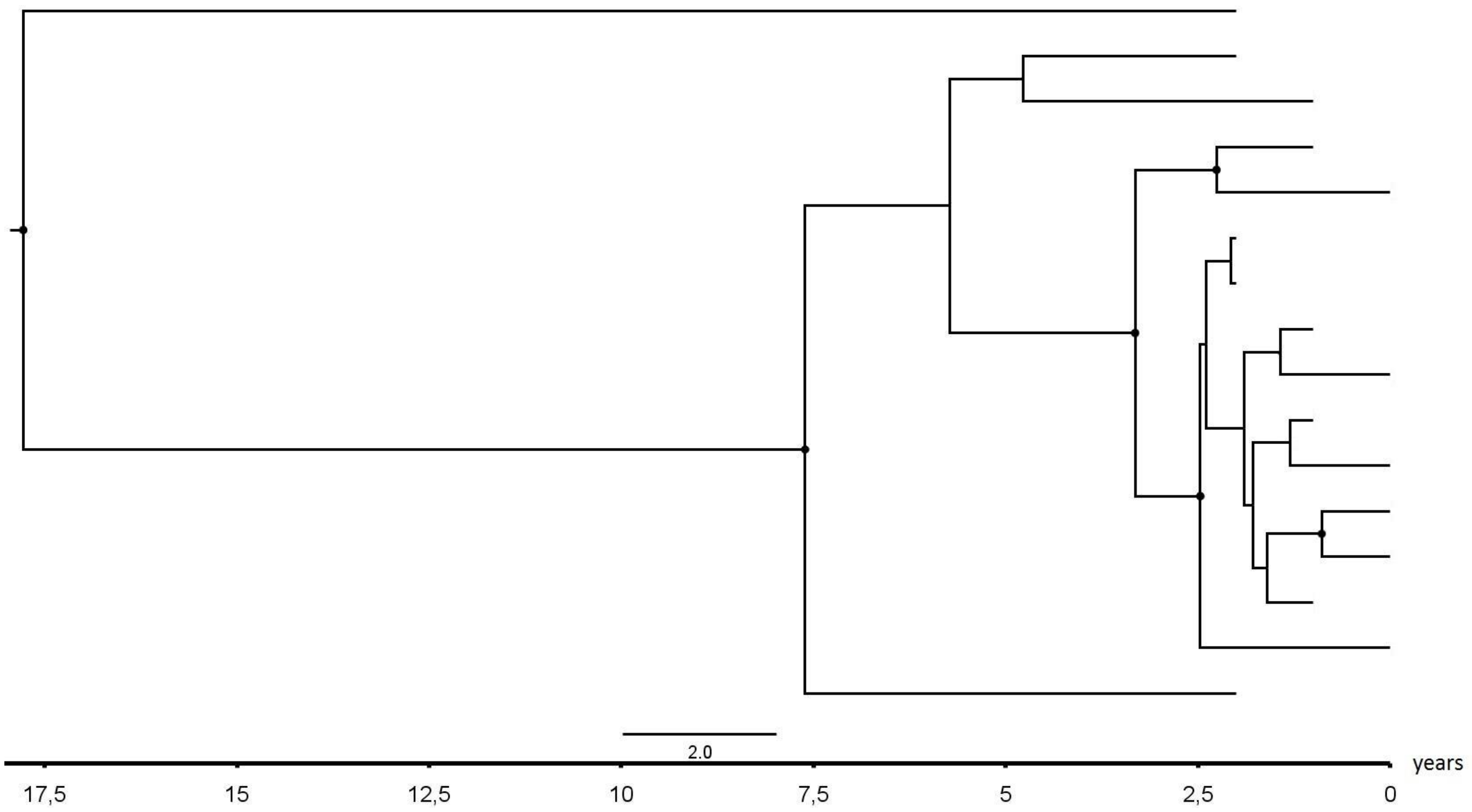

Supplementary Figure S.5 Cluster E

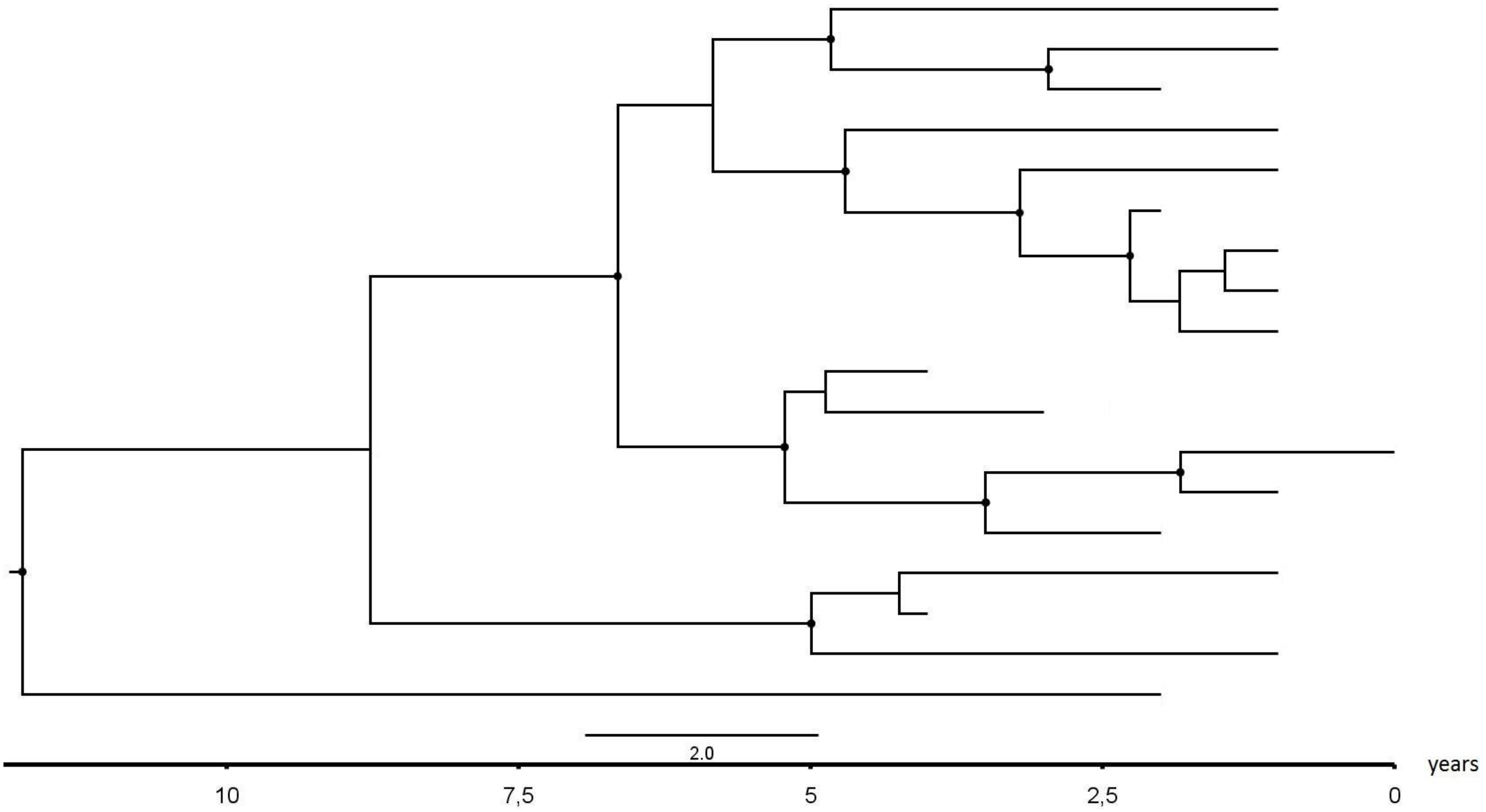

Supplementary Figure S.6 Cluster F

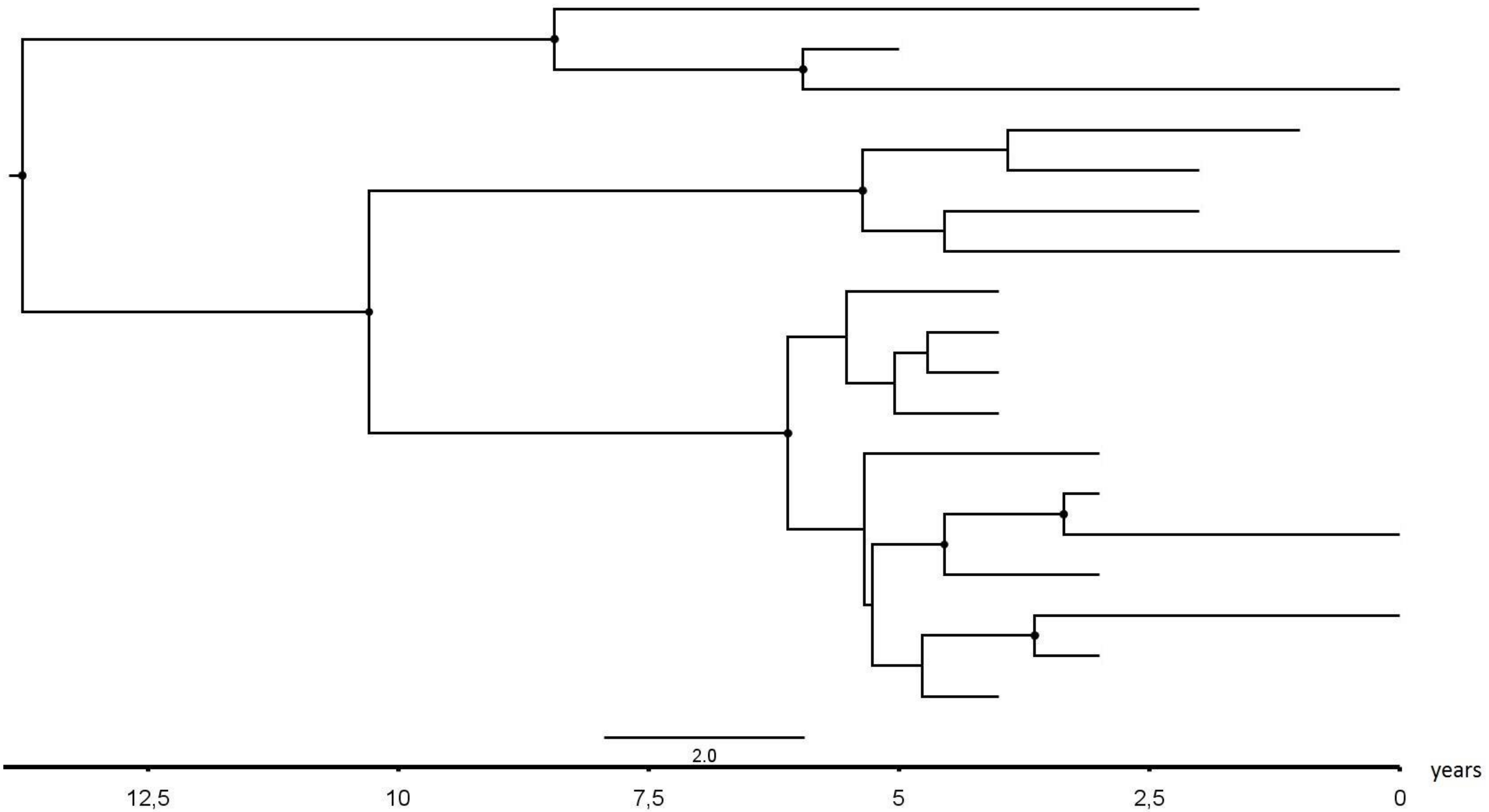

Supplementary Figure S.7 Cluster G

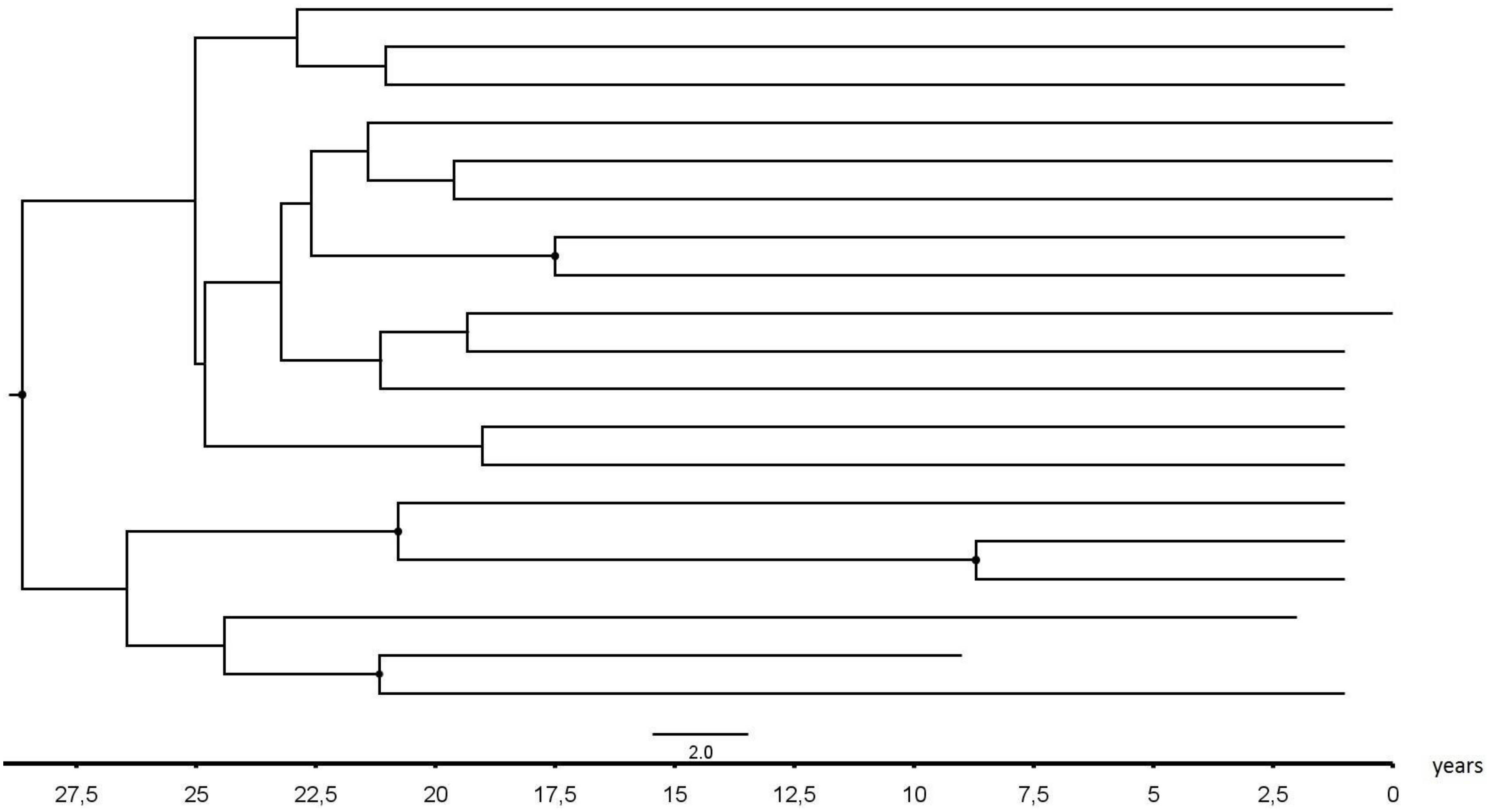

Supplementary Figure S.8 Cluster H

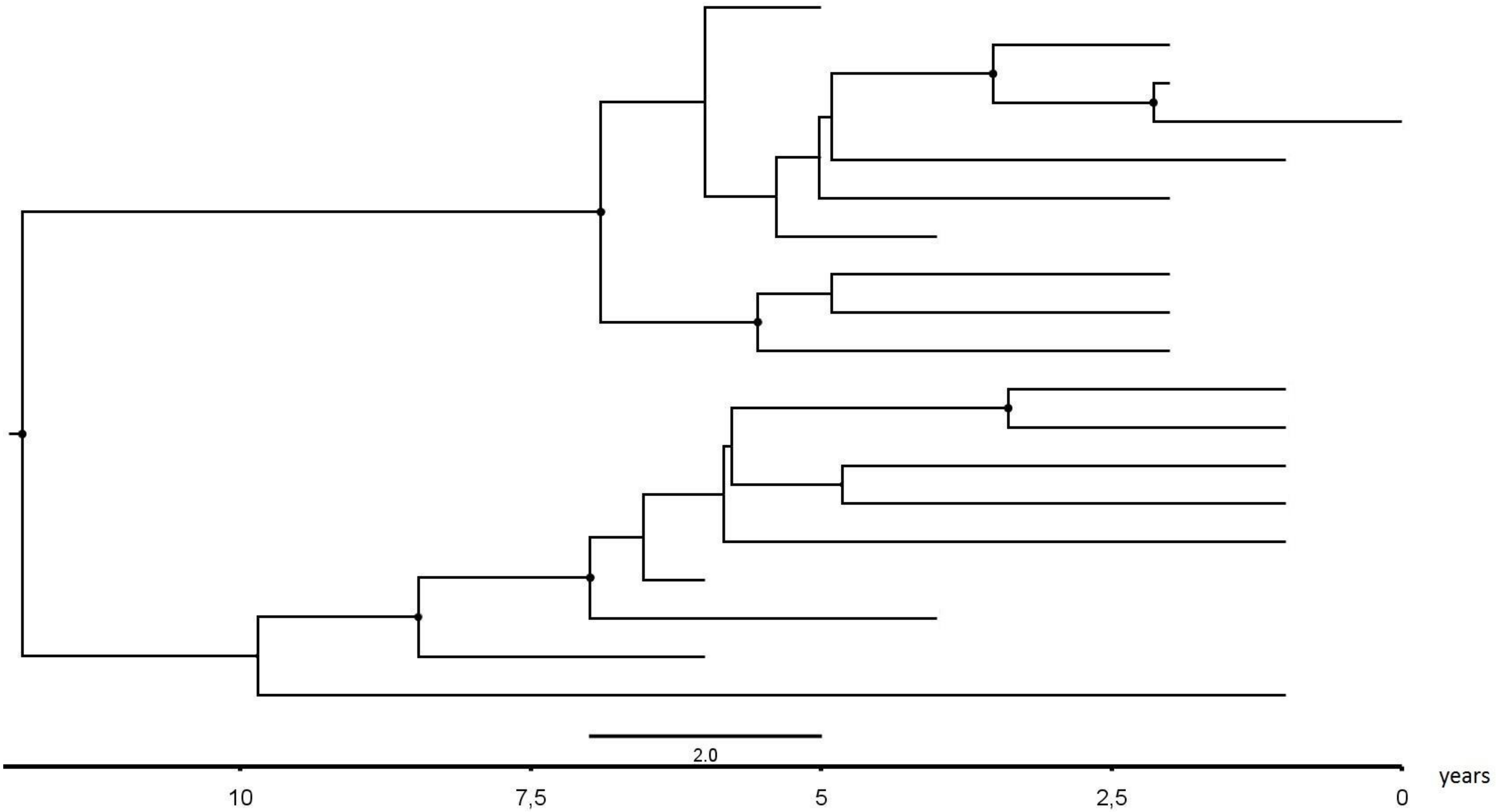

Supplementary Figure S.9 Cluster I

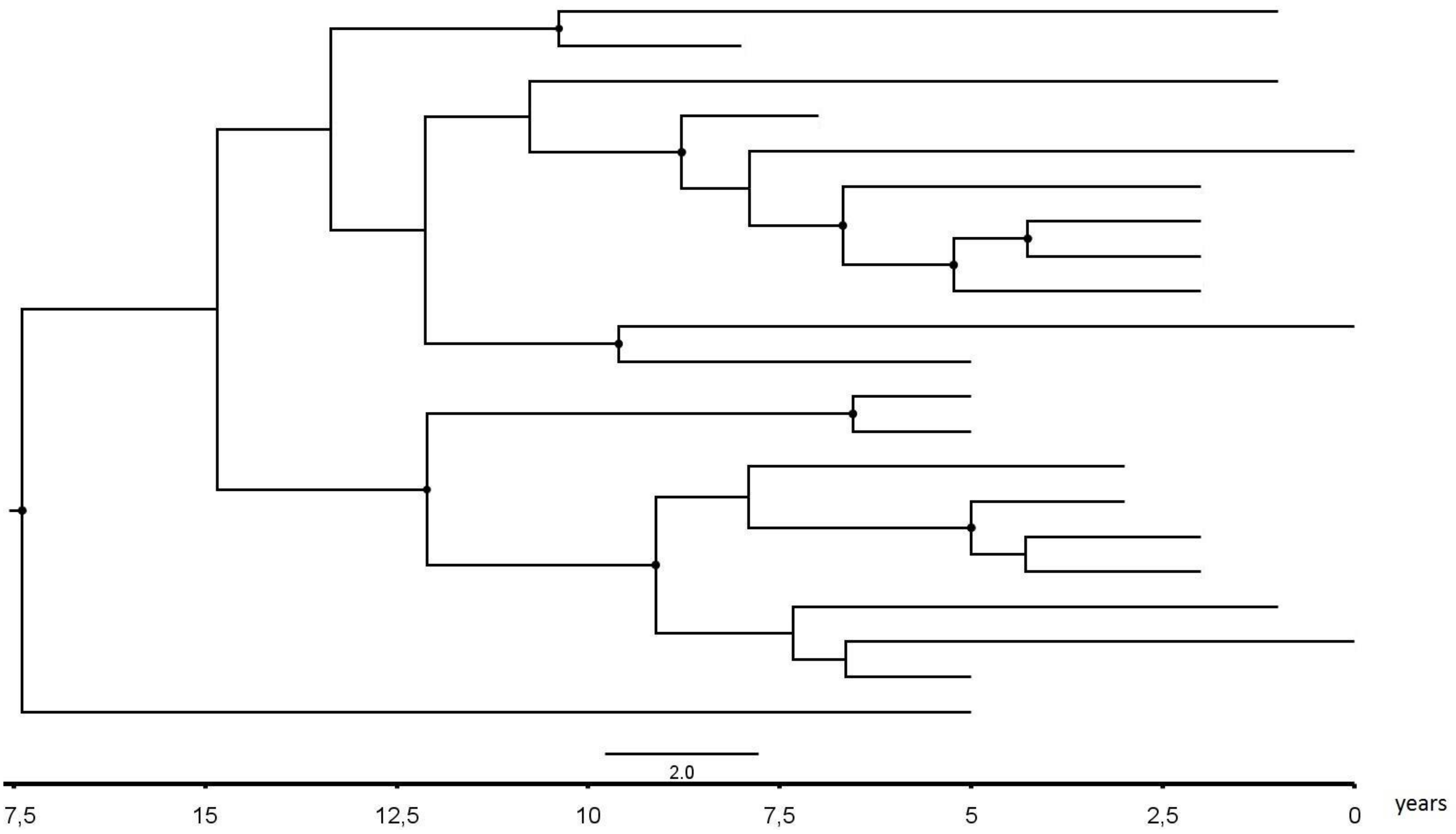

Supplementary Figure S.10 Cluster J

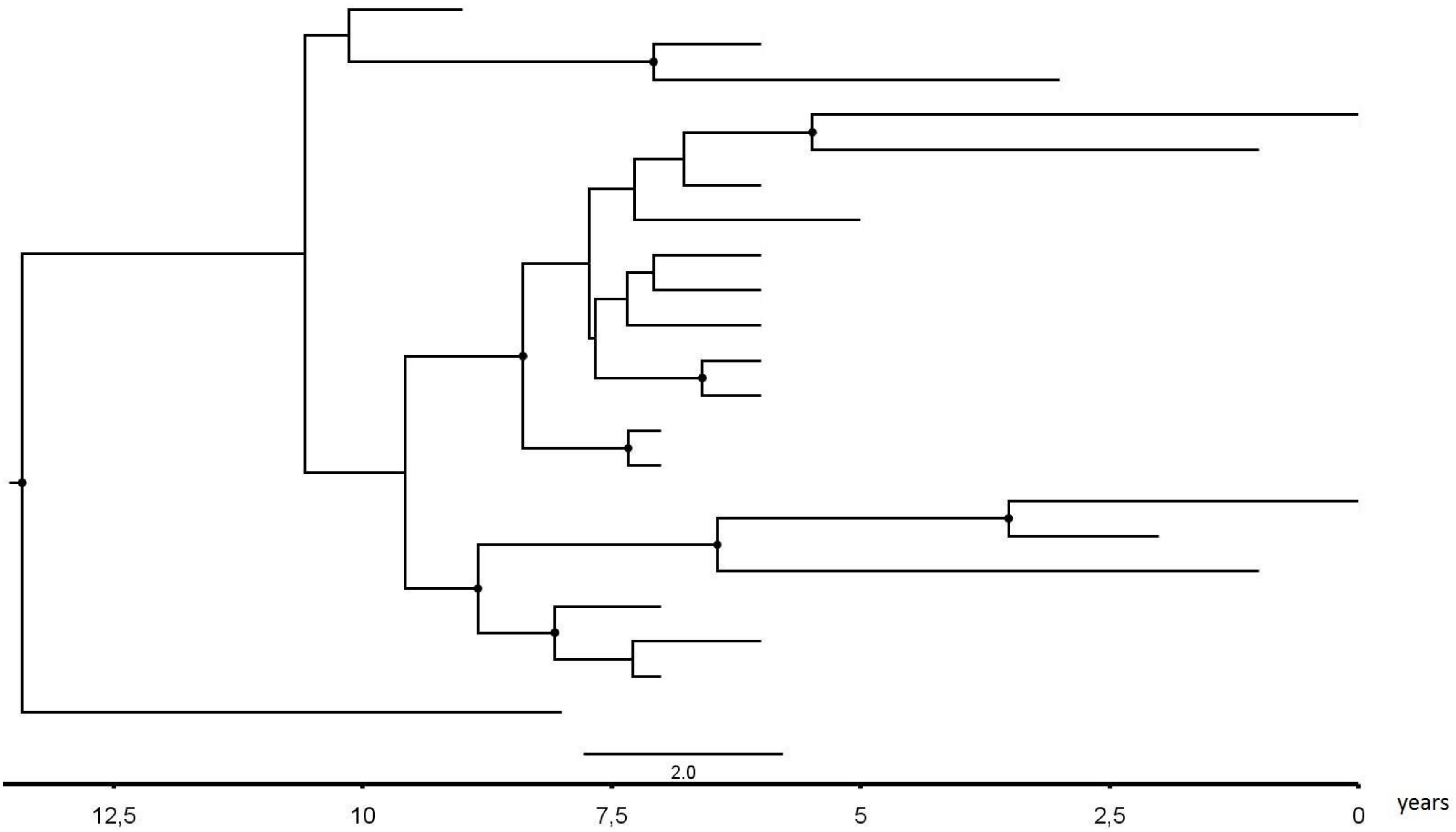

Supplementary Figure S.11 Cluster K

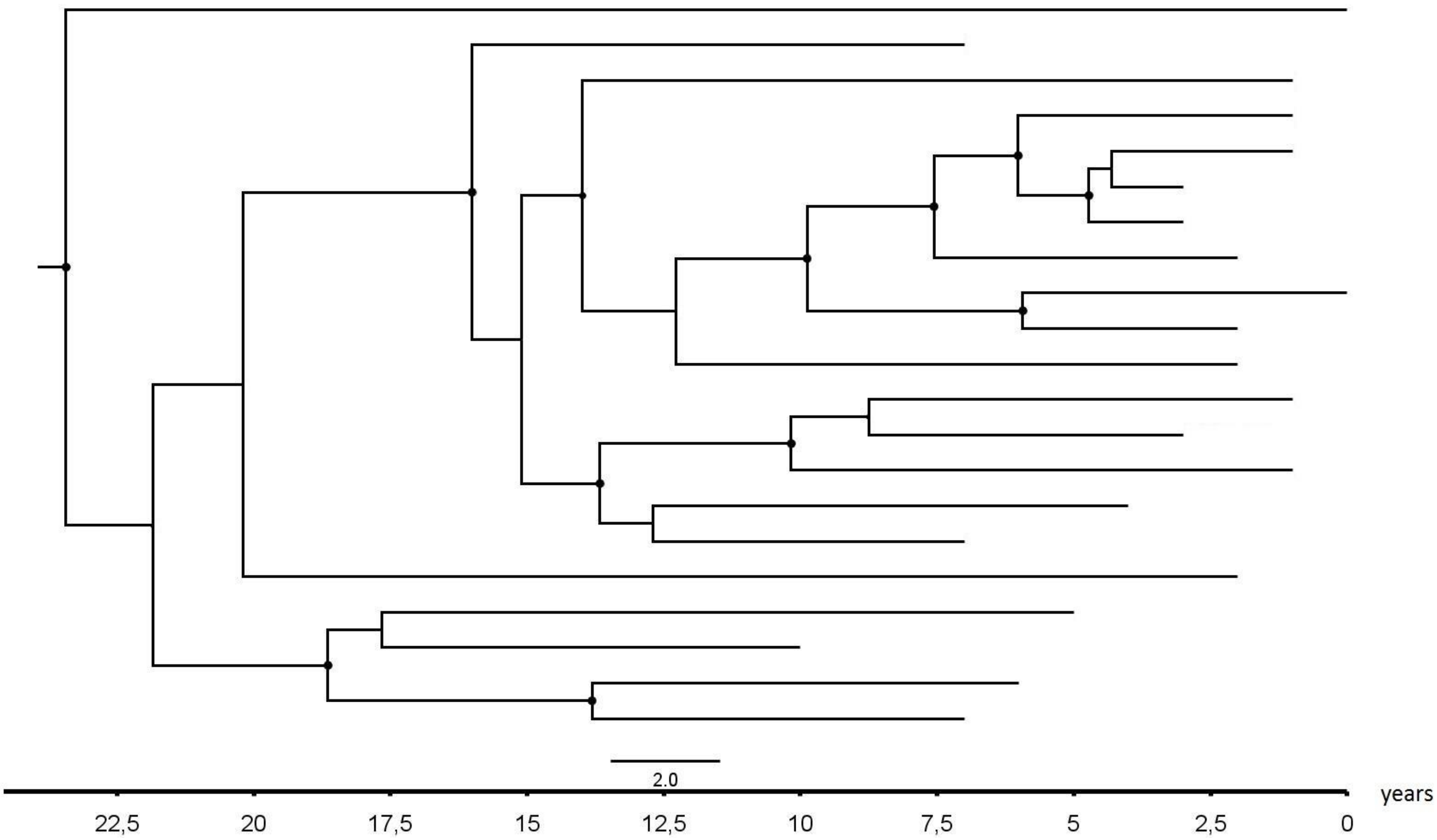

Supplementary Figure S.12 Cluster L

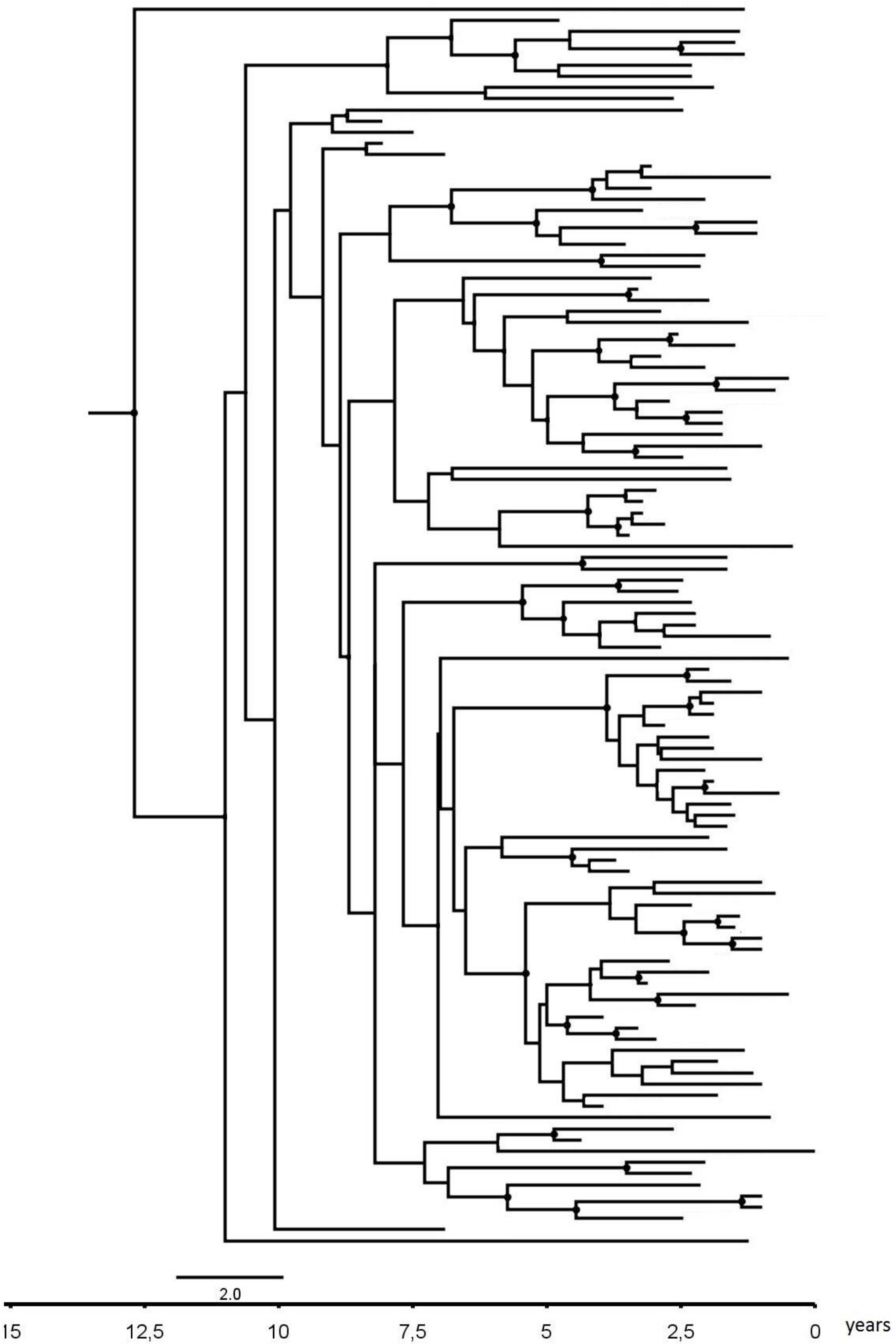

Supplementary Figure S.13

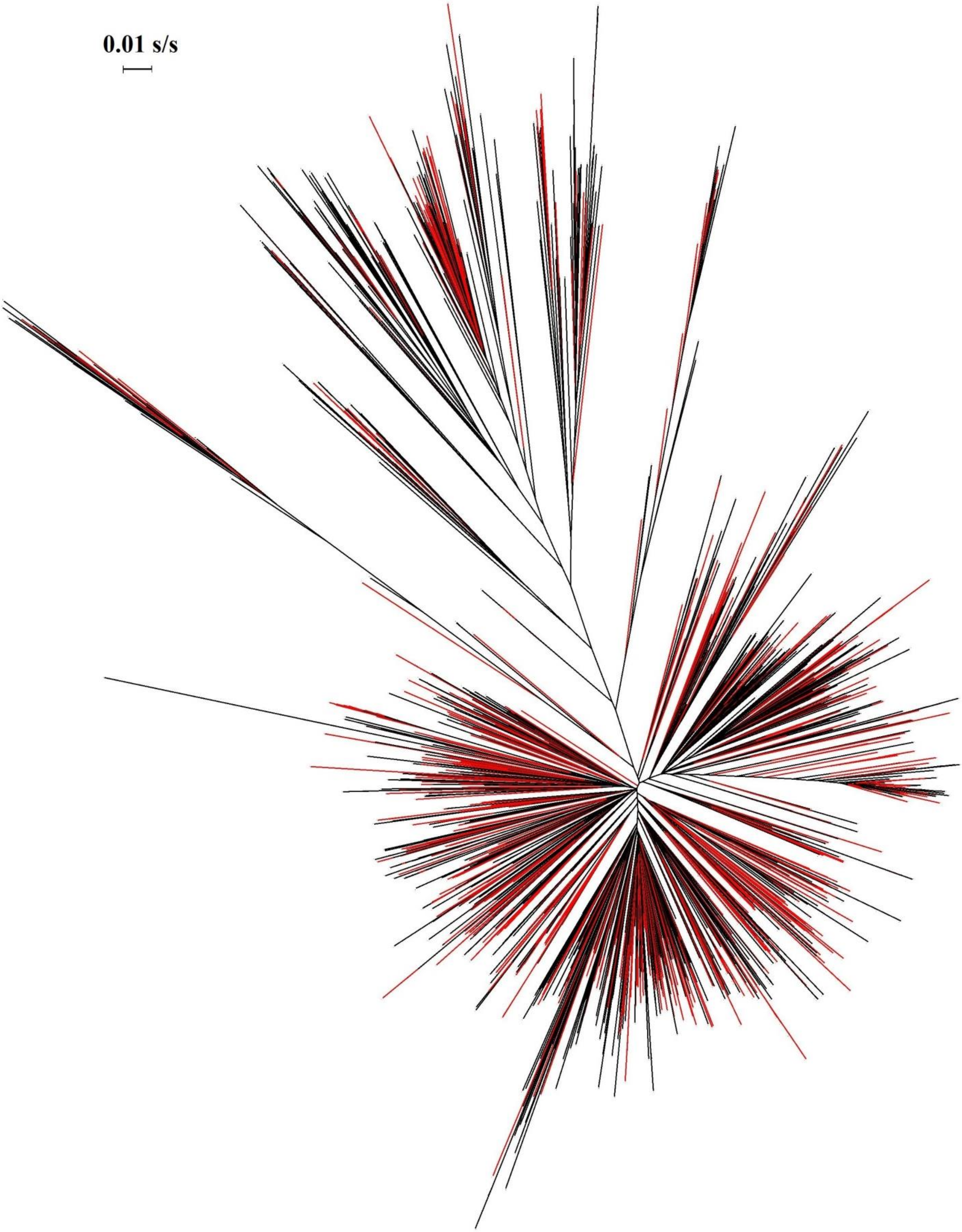

Supplementary Table S1. Geographical origin of patients from transmission clusters: Number of clusters, and number of patients (Spanish, non-Spanish, unknown origin), regarding the total number of B and non-B transmission cluste

		Spanish patients	non-Spanish patients	patients of unknown origin
**Total sequences clustering (subtype B): n= 219 clusters**		378	114 (W. Europe 20, E. Europe 10, Africa 10, L. America 73, Other 1)	400
**Total sequences clustering (no-B): n= 51 clusters**		31	47 (W. Europe 0, E. Europe 8, Africa 18, L. America 19, Other 2)	69
**Large clusters (n≥10 patients): n= 12 clusters**				
	A	6	2 (W. Europe 0, E. Europe 1, Africa 0, L. America 1, Other 0)	2
	B	5	3 (W. Europe 0, E. Europe 1, Africa 0, L. America 2, Other 0)	5
	C	3	3 (W. Europe 1, E. Europe 0, Africa 2, L. America 0, Other 0)	9
	D	8	2 (W. Europe 0, E. Europe 0, Africa 0, L. America 2, Other 0)	6
	E	5	2 (W. Europe 0, E. Europe 0, Africa 0, L. America 2, Other 0)	11
	F	12	4 (W. Europe 1, E. Europe 0, Africa 0, L. America 3, Other 0)	2
	G	2	0	17
	H	9	2 (W. Europe 0, E. Europe 0, Africa 0, L. America 2, Other 0)	8
	I	17	1 (W. Europe 1, E. Europe 0, Africa 0, L. America 0, Other 0)	3
	J	12	3 (W. Europe 1, E. Europe 0, Africa 0, L. America 2, Other 0)	6
	K (CRF14_BG)	7	4 (W. Europe 0, E. Europe 3, Africa 0, L. America 1, Other 0)	10
	L	53	15 (W. Europe 4, E. Europe 0, Africa 2, L. America 8, Other 1)	43
**Other clusters (mixed origin, subtype B): n = 38 cluster**		70	50 (W. Europe 7, E. Europe 4, Africa 4, L. America 35, Other 0)	18
**Other clusters (mixed origing subtypes no-B): n= 6 clusters**	(other subtype/CRF, MSM)	2	1 (W. Europe 0, E. Europe 0, Africa 0, L. America 1, Other 0)	1
	(subtype A, MSM)	1	1 (W. Europe 0, E. Europe 1, Africa 0, L. America 0, Other 0)	0
	(CRF02_AG, MSM)	1	1 (W. Europe 0, E. Europe 0, Africa 0, L. America 1, Other 0)	0
	(CRF02_AG, MSM)	3	1 (W. Europe 0, E. Europe 0, Africa 0, L. America 1, Other 0)	0
	(CRF02_AG, MSM)	2	1 (W. Europe 0, E. Europe 0, Africa 1, L. America 0, Other 0)	0
	(CRF19, MSM)	2	1 (W. Europe 0, E. Europe 0, Africa 0, L. America 1, Other 0)	2
**Other clusters (only Spanish origin, subtype B) : n= 88 clusters**		176	0	105
**Other clusters (only Spanish origin, subtypes no B): n= 9 clusters**		13	0	14
**Other clusters (only non-Spanish origin, subtype B): n= 16 clusters**		0	27 (W. Europe 4, E. Europe 5, Africa 2, L. America 16, Other 0)	16
**Other clusters (only non-Spanish origin, subtypes no-B): n= 25 clusters**		0	37 (W. Europe , E. Europe 4, Africa 17, L. America 14, Other 2)	20
**Other clusters (only unknown origin, subtype B): n=66 clustesr**		0	0	149
**Other clusters (only unknown origin, subtypes no-B): n= 10 clusters**		0	0	22

Supplementary Table S2. Cluster size distribution, if only patients included in the multinomial analysis were considered.

Cluster size	Number of clusters (B)	Number of clusters (Non-B)	Total number of patients	Number of patients (B)	Number of patients (B)
2	50	16	132	100	32
3	22	3	75	66	9
4	12	1	52	48	4
5	5		25	25	0
6	3		18	18	0
7	2		14	14	0
8	3		24	24	0
9	1		9	9	0
10		1	10	0	10
11	1		11	11	0
15	1		15	15	0
16	1		16	16	0
17	1		17	17	0
67	1		67	67	0
Not in clusters			421		

Supplementary Table S3. Summary of nucleotide divergence estimates among sequences within each cluster. Average, minimum and maximum values of Tamura-Nei estimates of nucleotide divergence

size	mean	min	max	min for category	max for category
2	0,01407932	0,01407932	0,01407932		
2	0,02037691	0,02037691	0,02037691		
2	0,00311974	0,00311974	0,003119742		
2	0,00465543	0,00465543	0,004655433		
2	0,00309418	0,00309418	0,003094179		
2	0,00077116	0,00077116	0,000771163		
2	0,01555741	0,01555741	0,01555741		
2	0,01089052	0,01089052	0,01089052		
2	0,00076998	0,00076998	0,000769978		
2	0,00309323	0,00309323	0,00309323		
2	0,00706134	0,00706134	0,007061335		
2	0,05846517	0,05846517	0,05846517		
2	0,01749437	0,01749437	0,01749437		
2	0,01330664	0,01330664	0,01330664		
2	0,05418696	0,05418696	0,05418696		
2	0,04843055	0,04843055	0,04843055		
2	0,00855739	0,00855739	0,008557393		
2	0,00394537	0,00394537	0,003945365		
2	0,0335854	0,0335854	0,0335854		
2	0,02089925	0,02089925	0,02089925		
2	0,00155856	0,00155856	0,001558557		
2	0	0	0		
2	0,05020592	0,05020592	0,05020592		
2	0,00388996	0,00388996	0,003889956		
2	0,03801678	0,03801678	0,03801678		
2	0,02941968	0,02941968	0,02941968		
2	0,02634543	0,02634543	0,02634543		
2	0,01102041	0,01102041	0,01102041		
2	0,00776672	0,00776672	0,007766715		
2	0,01271919	0,01271919	0,01271919		
2	0	0	0		
2	0,00779887	0,00779887	0,007798865		
2	0,00464182	0,00464182	0,004641817		
2	0,04953835	0,04953835	0,04953835		
2	0,0069567	0,0069567	0,0069567		
2	0,02209751	0,02209751	0,02209751		
2	0,00231668	0,00231668	0,002316678		
2	0,00076939	0,00076939	0,000769389		
2	0,00541245	0,00541245	0,005412447		
2	0,01402735	0,01402735	0,01402735		
2	0	0	0		
2	0,00386915	0,00386915	0,003869152		
2	0,01499798	0,01499798	0,01499798		
2	0,00782724	0,00782724	0,007827242		
2	0,0077603	0,0077603	0,007760299		

2	0,01327933	0,01327933	0,01327933
2	0,00386038	0,00386038	0,003860377
2	0,01013303	0,01013303	0,01013303
2	0,01570656	0,01570656	0,01570656
2	0,0047613	0,0047613	0,004761299
2	0,04171031	0,04171031	0,04171031
2	0,02371865	0,02371865	0,02371865
2	0,01021672	0,01021672	0,01021672
2	0,03537752	0,03537752	0,03537752
2	0,0180605	0,0180605	0,0180605
2	0,00622819	0,00622819	0,006228189
2	0,00697066	0,00697066	0,006970655
2	0	0	0
2	0,00852143	0,00852143	0,008521428
2	0	0	0
2	0,01492422	0,01492422	0,01492422
2	0,00386889	0,00386889	0,003868893
2	0,04375912	0,04375912	0,04375912
2	0,00232503	0,00232503	0,002325026
2	0,03188207	0,03188207	0,03188207
2	0,04285331	0,04285331	0,04285331
2	0,03042669	0,03042669	0,03042669
2	0,03914677	0,03914677	0,03914677
2	0,0456417	0,0456417	0,0456417
2	0,04957023	0,04957023	0,04957023
2	0,04609829	0,04609829	0,04609829
2	0,00462736	0,00462736	0,004627363
2	0,03261305	0,03261305	0,03261305
2	0,03198434	0,03198434	0,03198434
2	0,00388468	0,00388468	0,003884679
2	0,01182688	0,01182688	0,01182688
2	0,00780057	0,00780057	0,007800571
2	0,01572075	0,01572075	0,01572075
2	0,03010668	0,03010668	0,03010668
2	0,00790786	0,00790786	0,007907859
2	0,03624949	0,03624949	0,03624949
2	0,00233473	0,00233473	0,002334733
2	0,004688	0,004688	0,004687997
2	0,00234035	0,00234035	0,002340347
2	0,0023624	0,0023624	0,002362399
2	0,00077354	0,00077354	0,000773543
2	0,0038888	0,0038888	0,003888801
2	0,00154503	0,00154503	0,001545025
2	0,04631656	0,04631656	0,04631656
2	0,03186003	0,03186003	0,03186003
2	0	0	0
2	0,03228531	0,03228531	0,03228531
2	0,04399736	0,04399736	0,04399736
2	0,03034929	0,03034929	0,03034929
2	0,02219034	0,02219034	0,02219034

2	0,00930537	0,00930537	0,009305368
2	0,02853381	0,02853381	0,02853381
2	0,00386838	0,00386838	0,00386838
2	0,0095153	0,0095153	0,009515303
2	0,00154173	0,00154173	0,001541725
2	0,00232377	0,00232377	0,002323769
2	0,01263718	0,01263718	0,01263718
2	0,03043731	0,03043731	0,03043731
2	0,00465588	0,00465588	0,004655875
2	0,00703499	0,00703499	0,007034985
2	0,03704186	0,03704186	0,03704186
2	0,00931357	0,00931357	0,00931357
2	0,00231498	0,00231498	0,002314979
2	0,01815591	0,01815591	0,01815591
2	0,00856062	0,00856062	0,008560622
2	0,01326703	0,01326703	0,01326703
2	0,03858275	0,03858275	0,03858275
2	0,00541536	0,00541536	0,005415358
2	0,00547789	0,00547789	0,005477888
2	0,00233603	0,00233603	0,002336025
2	0,03956575	0,03956575	0,03956575
2	0,00077161	0,00077161	0,000771609
2	0,0103639	0,0103639	0,0103639
2	0,00308468	0,00308468	0,003084684
2	0,06199086	0,06199086	0,06199086
2	0,01088867	0,01088867	0,01088867
2	0,00697516	0,00697516	0,006975163
2	0,02363531	0,02363531	0,02363531
2	0	0	0
2	0,00385713	0,00385713	0,00385713
2	0,01334165	0,01334165	0,01334165
2	0,01178319	0,01178319	0,01178319
2	0,00864435	0,00864435	0,008644351
2	0,00718951	0,00718951	0,007189514
2	0,01504995	0,01504995	0,01504995
2	0,05754259	0,05754259	0,05754259
2	0,02060701	0,02060701	0,02060701
2	0,0054362	0,0054362	0,005436199
2	0,04348655	0,04348655	0,04348655
2	0,02941936	0,02941936	0,02941936
2	0,00077594	0,00077594	0,000775943
2	0,00154532	0,00154532	0,001545324
2	0,00699813	0,00699813	0,006998133
2	0,02303767	0,02303767	0,02303767
2	0,02380468	0,02380468	0,02380468
2	0,00154058	0,00154058	0,001540581
2	0,00154383	0,00154383	0,001543831
2	0,00312826	0,00312826	0,003128258
2	0,00697529	0,00697529	0,006975293
2	0,0117123	0,0117123	0,0117123

2	0,05197176	0,05197176	0,05197176		
2	0,01650465	0,01650465	0,01650465		
2	0,00857911	0,00857911	0,008579106		
2	0,0070578	0,0070578	0,007057797		
2	0,00621231	0,00621231	0,006212308		
2	0,00388128	0,00388128	0,003881276		
2	0,00076864	0,00076864	0,00076864		
2	0,00619952	0,00619952	0,006199524		
2	0,00784117	0,00784117	0,007841168		
2	0,02549561	0,02549561	0,02549561		
2	0,00856705	0,00856705	0,008567048		
2	0,00388569	0,00388569	0,003885692	0	0,06199086
3	0,0063239	0,00473099	0,009506314		
3	0,01658064	0,00940986	0,02371332		
3	0,02174038	0,01749957	0,0271881		
3	0,0043831	0,0038644	0,004642898		
3	0,00309104	0,00076937	0,004640847		
3	0,01148325	0,00935724	0,01334585		
3	0,03197479	0,01964227	0,04523888		
3	0,00622039	0,00308239	0,009359355		
3	0,04296876	0,03075738	0,05428724		
3	0,00992802	0,00864353	0,01090727		
3	0,02806742	0,0230566	0,03732714		
3	0,00103652	0	0,00155659		
3	0,01842511	0,01661295	0,01964292		
3	0,00678948	0,00548648	0,008640192		
3	0,0036229	0,0015498	0,00621546		
3	0,02716179	0,01897686	0,0370475		
3	0,00361836	0,00153861	0,004659764		
3	0,0069843	0,00464277	0,008545227		
3	0,01261373	0,00618927	0,0174333		
3	0,02978008	0,02381996	0,03778041		
3	0,0101379	0,00386068	0,01406879		
3	0,01360673	0,0085482	0,01733708		
3	0,01416323	0,00385997	0,02133342		
3	0,00620817	0,00154375	0,009326256		
3	0,03481387	0,00697045	0,04879494		
3	0,01072105	0,00309563	0,01492507		
3	0,00206146	0	0,003867735		
3	0,01099058	0,00859957	0,01334489		
3	0,01366454	0,00077102	0,02050768		
3	0,00673814	0,00231858	0,009338785		
3	0,00915402	0,00787668	0,01092457		
3	0,03749744	0,00780031	0,054799		
3	0,04287248	0,03339616	0,04927762		
3	0,01039021	0,0061963	0,01564698		
3	0,00678798	0,00231313	0,009432184		
3	0,00206189	0,000774	0,00386252		
3	0,00205815	0,00154218	0,00308695		
3	0,03834612	0,03023429	0,04527805		

3	0,04115188	0,03965025	0,04296907		
3	0,04116187	0,03532511	0,04591636		
3	0,00336553	0,00310325	0,003882219		
3	0,0307828	0,02188422	0,03817033		
3	0,03779083	0	0,05668625		
3	0,037446	0,03355573	0,044482		
3	0,00310458	0	0,004658453		
3	0,00180408	0,00077236	0,00309289		
3	0,00776374	0,00386963	0,01087206		
3	0,00542818	0,00077058	0,007756979		
3	0,00257829	0,00154297	0,003875168	0	0,05668625
4	0,03302255	0,01897758	0,04487088		
4	0,04183488	0,00155219	0,05404865		
4	0,00897971	0,00154502	0,01173318		
4	0,03317728	0,02245507	0,04557033		
4	0,01958587	0,00077476	0,03340481		
4	0,02142812	0,00545031	0,03605978		
4	0,04083185	0,00076997	0,06120187		
4	0,02010816	0,00466071	0,03518363		
4	0,00871153	0,00077116	0,01325536		
4	0,01902668	0,010125	0,03007308		
4	0,0104369	0,00699679	0,01492995		
4	0,02097404	0,0078571	0,02625563		
4	0,03645271	0,01892974	0,04981759		
4	0,00857023	0,00231614	0,01407484		
4	0,01588627	0,0046489	0,0237956		
4	0,01900906	0,01492399	0,02457171		
4	0,00549976	0,00232879	0,008698281		
4	0,01833496	0,01347349	0,02616688		
4	0,01356298	0,01171128	0,0149249		
4	0,01870381	0,00546903	0,03296013	0,000769967	0,06120187
5	0,00611064	0	0,01171148		
5	0,02026552	0,00389018	0,04333544		
5	0,0080884	0,00076864	0,01326621		
5	0,01887194	0,00935837	0,02535131		
5	0,01286031	0,00231804	0,01977785		
5	0,04129306	0,01329533	0,05699851		
5	0,03151016	0,00154631	0,05982574		
5	0,00543275	0,00076864	0,01012609		
5	0,01513515	0,00854312	0,01890563		
5	0,03019813	0,01089175	0,04207154		
5	0,00878493	0,00309561	0,01336832		
5	0,0293174	0,00859797	0,05374126		
5	0,01036745	0,00622266	0,01642578		
5	0,02620329	0	0,03959442		
5	0,01234583	0,00231089	0,01720033		
5	0,0431114	0,02299139	0,05832709	0	0,05982574
6	0,01902311	0,00626693	0,03042077		
6	0,05491786	0,00463787	0,073034		
6	0,03938926	0,0077698	0,06418437		

6	0,01834783	0,0055764	0,03015163	0,004637874	0,073034
7	0,02685159	0,01246852	0,04934855		
7	0,02569554	0,00542307	0,04343757		
7	0,01602924	0,00864681	0,02239488		
7	0,00682945	0	0,01561948		
7	0,00905077	0,0030908	0,01642189		
7	0,00814073	0,00463734	0,0133149	0	0,04934855
8	0,04885219	0,01094283	0,06946943		
8	0,02318093	0	0,03528201		
8	0,00387028	0	0,006986901		
8	0,02286688	0,00386653	0,03448203	0	0,06946943
9	0,01453208	0,00077058	0,03451436		
9	0,01302328	0,00231128	0,02377225	0,000770584	0,03451436
10	0,04223366	0,00154293	0,06013774	0,00154293	0,06013774
13	0,02036827	0,00076982	0,0369425	0,000769823	0,0369425
15	0,03829381	0	0,06406709	0	0,06406709
16	0,01817182	0	0,0679065	0	0,0679065
18	0,01652194	0	0,03707785		
18	0,01642938	0,00153934	0,0366595	0	0,03707785
19	0,04306046	0,0109264	0,06940829		
19	0,02229143	0,00076998	0,04352066	0,000769976	0,06940829
21	0,02091908	0,00311059	0,03685226		
21	0,0153959	0,0007694	0,03524647		
21	0,02526316	0,00389022	0,04803633	0,000769399	0,04803633
111	0,0181625	0	0,04096108	0	0,04096108

Supplementary Table S4. Distribution of HIV cases in the dataset subjected to multinomial analysis (n=906), regarding the variables subtype, gender, age, nationality, risk group and clustering status

	B (n=752)	A1 (n=14)	F1 (n=13)	G (n=11)	CRF02_AG (n=42)	CRF14_BG (n=15)	Others (n=59)	Total (n=906)
**Gender**								
Male	672	9	8	4	22	9	41	765
Female	80	5	5	7	20	6	18	141
**Age**								
<=20	30	1	2	0	1	0	1	35
21-29	246	5	3	9	24	2	25	314
30-35	194	4	2	1	9	6	14	230
36-50	252	3	6	1	8	4	18	292
>50	30	1	0	0	0	3	1	35
**Nationality**								
Spain	555	6	4	3	11	8	20	607
W.Europe and N America	27	0	0	0	1	0	1	29
Eastern Europe	15	5	3	0	0	4	6	33
Africa and M. East	13	2	3	8	23	0	11	60
Latin America	141	1	3	0	7	3	20	175
Others	1	0	0	0	0	0	1	2
**Risk group**								
HT	119	8	7	9	29	2	24	198
MSM	564	3	6	0	11	1	30	615
IDU	67	3	0	2	2	12	5	91
Other	2	0	0	0	0	0	0	2
**Clustering**								
No	277	10	10	5	23	3	34	362
Small cluster (2-3)	170	4	1	6	14	2	18	215
Medium cluster (4-9)	142	0	2	0	5	0	7	156
Large cluster (>=10)	163	0	0	0	0	10	0	173
